# Quantum Chemical Modeling of Pressure‐Induced Spin Crossover in Octahedral Metal‐Ligand Complexes

**DOI:** 10.1002/cphc.201900853

**Published:** 2019-10-10

**Authors:** Tim Stauch, Romit Chakraborty, Martin Head‐Gordon

**Affiliations:** ^1^ University of Bremen Institute for Physical and Theoretical Chemistry Leobener Str. NW2 28359 Bremen Germany; ^2^ Kenneth S. Pitzer Center for Theoretical Chemistry Department of Chemistry University of California Berkeley California 94720 United States of America; ^3^ Materials Sciences Division Lawrence Berkeley National Laboratory Berkeley California 94720 United States of America; ^4^ Chemical Sciences Division Lawrence Berkeley National Laboratory Berkeley California 94720 United States of America

**Keywords:** density functional theory, metal−ligand complexes, pressure, quantum chemical modeling, spin crossover

## Abstract

Spin state switching on external stimuli is a phenomenon with wide applicability, ranging from molecular electronics to gas activation in nanoporous frameworks. Here, we model the spin crossover as a function of the hydrostatic pressure in octahedrally coordinated transition metal centers by applying a field of effective nuclear forces that compress the molecule towards its centroid. For spin crossover in first‐row transition metals coordinated by hydrogen, nitrogen, and carbon monoxide, we find the pressure required for spin transition to be a function of the ligand position in the spectrochemical sequence. While pressures on the order of 1 GPa are required to flip spins in homogeneously ligated octahedral sites, we demonstrate a fivefold decrease in spin transition pressure for the archetypal strong field ligand carbon monoxide in octahedrally coordinated Fe^2+^ in [Fe(II)(NH_3_)_5_CO]^2+^.

## Graphical abstract

**Molecules under pressure**: By using mechanical forces that compress a molecule towards its centroid, the hydrostatic pressure required for spin‐crossover is studied in a variety of transition‐metal complexes. Recommendations on the choice of ligands to achieve a desired spin transition pressure are given.

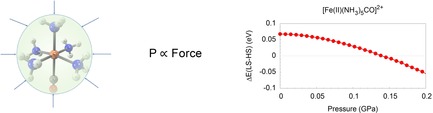

Pressure‐induced spin‐flips of transition metal sites involve changes in Coulomb energy, closed shell repulsions, covalent bonding energy and crystal field energy.^1^, ^2^ Using the computational Extreme‐Pressure PCM (XP‐PCM) protocol,^3^, ^4^, ^5^ it has been shown that high pressures cause drastic electronic rearrangements, causing first row transition metals with electronic configurations d^n^ (4≤n≤8) to favor low spin configurations at high pressures.^6^ In a metal‐organic framework (MOF) with exposed Fe(II) sites, a distinct step as a function of atmospheric pressure has been observed in the adsorption isotherm of carbon monoxide, for which a cooperative spin‐transition mechanism involving interacting iron centers in the MOF on introduction of the strong‐field ligand carbon monoxide has been proposed.^7^ In general, the use of MOFs in the separation of industrially relevant gases like hydrogen, nitrogen and carbon monoxide holds a lot of promise, due to the large surface areas, the thermal stability and the adjustability of various parameters of the MOFs.^8^

The smallest building block of spin crossover systems is often an octahedrally coordinated transition metal center with its 3d orbitals split by the ligand field environment. Spin‐flip at high pressures can be attributed to the increase in splitting of the 3d levels at the metal site such that the potential energy required to maintain a high spin configuration surpasses the spin pairing energy.^9^ Using effective nuclear forces scaled by their distances from the molecular centroid we here model spin crossover in octahedral metal‐ligand complexes as a function of hydrostatic pressure. We find the pressure required for spin transition to be a function of ligand position in the spectrochemical sequence^10^ and demonstrate that the spin transition pressure can be tuned by an adequate choice of the ligand field. Finer quantum chemical effects at play in the process involve a change in the covalent binding energy, Pauli repulsion, and charge transfer as a function of pressure, as demonstrated with an Energy Decomposition Analysis (EDA)^11^ scheme.

Any external force on atomic nuclei modifies the molecular potential energy surface (PES).^12^ Optimal molecular geometries under external forces can be computed with an assortment of techniques that yield a force‐modified potential energy surface (FMPES).^13^, ^14^, ^15^ These techniques quantify the change in the molecular PES under external stresses and the resulting changes in observables. Examples include shifts of signals in infrared^16^, ^17^, ^18^ and optical spectra,^18^, ^19^, ^20^ as well as changes in reaction kinetics.^21^, ^22^ Spatially varying nuclear forces have been described in a previous work as a generalized force‐modified potential energy surface (G‐FMPES).^23^ Hydrostatic pressure on a molecule can be modeled with such a field of effective nuclear forces that are scaled by their distances to the geometric center of the molecule.^23^, ^24^ For a spherical molecule, effective forces act perpendicular to the molecular surface, thereby leading to a compression mimicking the effect of hydrostatic pressure on the molecule. Equilibrium nuclear configurations for a given pressure are obtained when the restoring force of nuclei in the molecule equals the force due to an externally applied hydrostatic compression force field (HCFF) on nuclei during a geometry optimization.

In our HCFF algorithm presented here we make two departures from previous G‐FMPES algorithms.^23^, ^24^ First, starting from the definition of pressure, we estimate the maximum force, which acts on the outermost atom, as

(1) $$f_{max}=-P_{guess}\cdot A_{vdW}$$

Here, P_guess_ is a guess for the hydrostatic pressure, which is input by the user. We choose this definition of the force applied to the outermost atom because it involves the molecular van‐der‐Waals surface A_vdW_, which constitutes the interface between the molecule and the environment through which hydrostatic pressure is applied to the molecule. The negative sign ensures that all forces are compressive and directed inward. The molecular surface area is computed by numerically integrating the molecular van‐der‐Waals surface tessellated with a Lebedev grid.^25^, ^26^ The force acting on each atom i is then evaluated as

(2) $$f_{i}=f_{max}\cdot \frac{r_{i}}{r_{max}},$$

where r_max_ is the maximum distance of any atom to the geometric center, making sure that the largest force, f_max_, acts on the outermost atom. r_i_ is the distance of atom i from the molecular centroid. As shown previously, such a scaling guarantees that the force field is conservative.^23^ Thus, eq. 2 ensures that no net translation or rotation occurs in the molecule. Technically, the force f_i_ on each atom i is added to the nuclear gradient during a geometry optimization.

In the original G‐FMPES scheme the pressure is estimated by the ratio between the average force acting on the atoms, ⟨∥f∥⟩ , and the average area of spheres around the geometric center on which the atoms are placed.^24^ Since we define the force acting on the atoms via the molecular van‐der‐Waals surface (cf. eq. 1), we instead use the molecular van‐der‐Waals surface area for the calculation of the hydrostatic pressure:

(3) $$P_{HP}=\frac{\left\langle ∥f∥\right\rangle }{A_{vdW}}$$

All pressures reported in this paper refer to P_HP_. Since the forces on atoms closer to the molecular centroid are scaled down according to eq. 2, P_guess_ is typically an upper bound to P_HP_. Further computational details can be found in the Supporting Information.

Since hydrostatic pressure in this scheme is related to the molecular surface area, an accurate numerical estimate of molecular surface area is critical for computing the effective hydrostatic pressure. Our algorithm based on numerical integration of the molecular van‐der‐Waals surface on a tightly‐defined Lebedev grid^25^, ^26^ serves that purpose. With this, an effective hydrostatic compression force field (HCFF) envelopes a molecule, enabling us to optimize the geometry of a molecule under a given hydrostatic pressure. In the present paper, we use this methodology to determine the preferred electronic configuration of octahedral metal‐ligand complexes as well as the transition pressure. It should not be forgotten that all forces point towards the centroid of the molecule under consideration, which means that the protocol works best for molecules with a spherical symmetry (Figure 1). As one would expect in such model spherical approximations, there are in some cases deviations from octahedral symmetry at high pressures that may exhibit themselves as kinks in otherwise smooth curves. We elaborate on such fluctuations in the Supporting Information. Other recipes for simulating hydrostatic pressure are conceivable. For periodic systems, procedures are available that simulate pressure by modulating lattice parameters.

**Figure 1:**
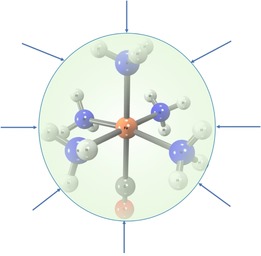
The Hydrostatic Compression Force Field (HCFF) compresses a molecule uniformly towards its centroid, with a scalar (P_guess_) that determines the magnitude of hydrostatic pressure (P_HP_) acting on it.

To validate the HCFF model we compared the bond length and total energy of H_2_^+^ as a function of pressure against reference values by Gorecki and Byers Brown,^27^ who calculated these quantities up to approx. 10^6^ bar (100 GPa) with iterative boundary perturbation theory in a hard spheroidal box. We applied the HCFF method at the Hartree‐Fock/cc‐pVTZ^28^ level of theory, due to the lack of electron correlation and the very good agreement of the bond length at P=0 (1.998 a.u.) to the literature value (2.000 a.u.). The results are given in Figure 2.

**Figure 2:**
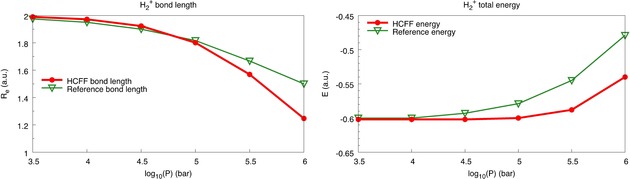
Bond length (left) and total energy (right) of H_2_^+^ as a function of pressure, as calculated with the HCFF method (red dots). Reference values (green triangles) were taken from ref. [27].

Focusing on the bond length of H_2_^+^ as a function of pressure first, one can observe that up to 10^5^ bar the HCFF method agrees remarkably well with the results by Gorecki and Byers Brown. At pressures larger than 10^5^ bar the bond length decreases more rapidly than the reference values. However, the spin transition pressures calculated for the octahedral metal‐ligand complexes reported in this paper are well below 10^5^ bar (10 GPa), so the correct reproduction of the geometry in H_2_^+^ is a valuable result.

Conversely, the increase in energy of H_2_^+^ with increasing pressure is less pronounced in HCFF than in iterative boundary perturbation theory. At 10^5^ bar the difference between HCFF and the literature value is approx. 0.02 a.u., notwithstanding the excellent agreement between the bond lengths at this pressure. To rationalize this effect it is important to recall that in the HCFF model forces are applied to the nuclei and the electron density is not constrained or compressed in any way other than indirectly through an altered nuclear configuration. The observed differences in energies may therefore be attributed to the lack of kinetic energy compression. We acknowledge this lack of a contribution from kinetic energy compression as a limitation in our model. For a wavefunction in a cavity, the kinetic energy is an important contributor to pressure‐induced energy changes. However, buried deep within the octahedral complex, electrons at the metal site are somewhat shielded from the effects of pressure applied at its boundaries. The extremal ligands, which are most affected by pressure applied on the boundary region, are identical for two spin states of a metal ion and we reason here that the kinetic energy compression may cancel out as we compute energy differences.

The ground spin state in first row transition metals is high spin as per Hund's rule of maximum multiplicity.^9^, ^29^ An octahedral ligand field splits the d manifold of the metal into t_2g_ and e_g_ orbitals. The energy difference between high and low spin states, an implicit function of the difference between the energies of the t_2g_ and e_g_ orbital sets, ▵_O_, can be modulated by the ligand field environment. Spin crossover occurs in transition metal complexes when the energy required to pair spins to reach a low spin configuration is offset by the cost of maintaining a high spin configuration due to an increase in ▵_O_. While various external stimuli can result in spin crossover in transition metal complexes, we focus here on spin crossover as a function of pressure.^30^ With a guiding hypothesis that uniform mechanical stress that compresses the molecule forces electron pairing and hence favors low spin states, we proceeded to apply hydrostatic pressure to first row transition metals with a homogeneous octahedral ligand field consisting of molecular hydrogen, nitrogen, and carbon monoxide, using the ωB97M‐V^31^/def2‐TZVP^32^ level of theory. While the results presented here were obtained with single reference Density Functional Theory (DFT),^33^, ^34^ the ωB97M‐V functional^31^ has proved very accurate both for main group^35^, ^36^ and transition metal^37^ chemical energy differences.

The quintessential strong‐field ligand carbon monoxide was found to already flip the spins in all but one metal ions in our dataset at P=0. Table 1 lists the spin gaps in eV of selected first row transition metal ions in the presence of a ligand field along with the pressure (in GPa) at which spin crossover occurs. Labelled in column 2 (M_hs_, M_ls_) are spin multiplicities (M_x_=2S_x_+1) for high and low spin configurations of transition metal ions for which we model a pressure‐induced spin crossover. Experimental spin gaps (eV) of bare metal ions obtained from the NIST atomic spectra database^38^ are listed in column 3. The total energies, <S^2^> values and geometries of the complexes at P=0 and the spin transition pressure are given in the Supporting Information.

**Table 1:** Multiplicities for the high spin and low spin configurations of metal ions as well as the corresponding spin gaps (taken from the NIST database).^38^ Spin gaps at P=0 and spin transition pressure for the high spin to low spin crossover are given for selected octahedral metal‐ligand complexes. Transition pressures were rounded to the nearest 0.05 GPa. For cases where no transition pressure is given, the low spin state is already energetically more favorable at P=0.

Metal	(M_hs_, M_ls_)	Spin Gap [eV]				Spin transition pressure [GPa]		
		Bare	CO	N_2_	H_2_	CO	N_2_	H_2_
Co^2+^	(4,2)	2.03	−0.20	0.76	0.62	–	1.40	2.20
Cr^2+^	(5,3)	2.11	−0.14	0.56	0.51	–	1.10	1.65
Fe^2+^	(5,1)	3.69	−1.14	0.58	0.44	–	0.70	1.20
Fe^3+^	(6,2)	5.84	−0.57	0.87	0.66	–	1.40	2.05
Mn^2+^	(6,2)	4.86	0.52	1.92	1.87	0.55	0.90	3.65

The position of the ligands in the spectrochemical series^10^ modulates the spin gap in octahedral metal‐ligand complexes as evidenced by their change in sign for the strong field ligand carbon monoxide in all but one instance, and diminution for metal ions coordinated with nitrogen and hydrogen. The spin gap for a metal in a given ligand field provides an estimate of the work required to affect spin state switching, and is influenced by its ligand field environment. For a given metal, the spin transition occurs at a lower pressure for a stronger field ligand. This may be readily explained by stronger splitting of the 3d levels at the metal site by a strong‐field ligand leading to a higher potential energy cost to maintain a high spin configuration. Turning to a comparison between the different metal sites, the results cannot be easily generalized. For instance the spin gap in [Mn(II)(N_2_)_6_]^2+^ (1.92 eV) is larger than in [Fe(III)(N_2_)_6_]^3+^ (0.87 eV), whereas the transition pressure in the former complex (0.90 GPa) is lower than in the latter (1.40 GPa). This highlights the specificity of metal‐ligand interactions in determining the spin transition pressure. All pressures reported in Table 1 are readily available in diamond anvil cells,^39^ thus providing the opportunity for experimental verification.

Following up on the validated hypotheses that spins pair under high pressures and that the ligand field environment influences the pressure required to flip metallic spins, we looked for candidate molecules that would show a spin transition at lower pressures. For gas separation in extended frameworks, spin transitions have been measured at close to ambient atmospheric pressures.^7^ A lower spin transition pressure is desirable since it can facilitate energy efficient gas separation. In a mixed ligand system where the Fe^2+^ cation is surrounded by five ammonia and one carbon monoxide molecules, the HCFF model predicts a spin crossover at approx. 0.14 GPa. Figure 3 compares the effect of pressure on the spin gap in Fe^2+^ in a homogenous ligand field with the weak field ligand H_2_, where it transitions at 1.2 GPa, to that in the mixed‐ligand system ([Fe(II)(NH_3_)_5_CO]^2+^. In both complexes, the energy difference between the high spin and low spin states as well as the average metal‐ligand distances decrease continuously. In a homogenous ligand field of carbon monoxide, the metal is already in the low spin configuration (see Table 1). However, on tuning the ligand field to consist of five weak‐field ammonia ligands and one carbon monoxide ligand, the ground state is kept at high spin (quintet). This provides an interesting opportunity to finely tune the ligand field around the metal ion to obtain a desirable spin transition pressure.

**Figure 3:**
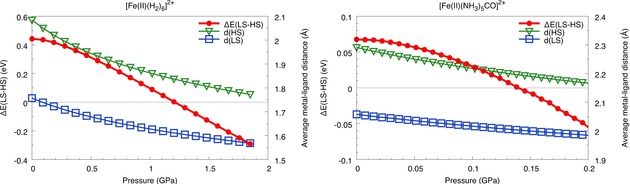
The effect of hydrostatic pressure on the spin gap of octahedrally coordinated Fe^2+^. For a charge neutral ligand field consisting i) of six hydrogen molecules ([Fe(II)(H_2_)_6_]^2+^, left), a spin transition occurs at 1.2 GPa, and ii) of five ammonia and one carbon monoxide molecules ([Fe(II)(NH_3_)_5_CO]^2+^, right), the low spin (singlet) state becomes energetically favored at an external pressure of approximately 0.14 GPa. The average metal‐ligand distance in the high spin (green triangles) and low spin (blue squares) states is given on the second y‐axis.

An investigation into electronic response to hydrostatic pressure may be carried out with systematic delineation of permanent electrostatics, Pauli repulsion and dispersion interactions from charge transfer with an Energy Decomposition Analysis (EDA) using Absolutely Localized Molecular Orbitals (ALMOs) based on a scheme detailed in Ref. [11]. The effect of pressure on spin gaps in the two systems in Figure 3 is thus complemented by a study of the different electronic effects at play in the process in Figure 4. We observe the expected linear increase in Pauli repulsion as a function of pressure (shown by steep positive slopes of ▵E_FRZ_). Charge transfer between the metal ion and the ligands is noticeably favored for low spin states at higher pressures. In contrast to Pauli repulsion, for which change with respect to pressure is almost identical for high and low spin configurations (notice similar slopes for high and low spin curves) the charge transfer term ▵E_VCT_ increases more rapidly for low spin states, making them more favored. This emphasizes the role of electronic orbital interactions in causing metal spins to flip at higher pressures.

**Figure 4:**
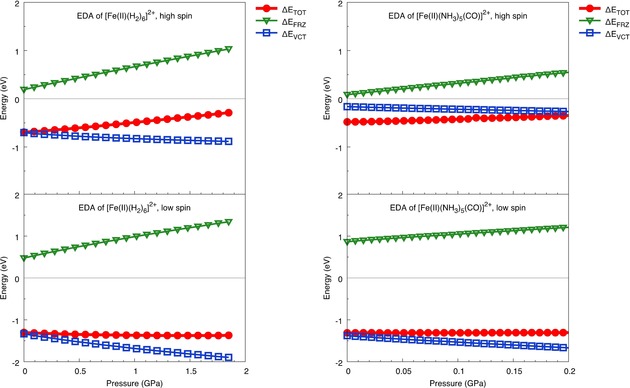
Energy Decomposition Analysis of contributions to total energy in high spin (top) and low spin (bottom) [Fe(II)(H_2_)_6_]^2+^ (left) and [Fe(II)(NH_3_)_5_CO]^2+^ (right). ▵E_TOT_ (red circles) refers to the total SCF energy, ▵E_FRZ_ (green triangles) to changes in Pauli repulsion and ▵E_VCT_ (blue squares) to charge transfer as a function of hydrostatic pressure.

The influence of the ligand field on a metal ion may drive electronic rearrangement in an open metal site and this effect is amplified by hydrostatic pressure. In octahedrally coordinated metal‐ligand complexes, we find that the required electron‐density reorganization can indeed be achieved by hydrostatic pressure, which we find to be typically on the order of 1 GPa (several thousand atm) for the complexes considered here. The onset of spin transition correlates with the position of the ligands in the spectrochemical series: In general, a stronger ligand field results in a lower spin transition pressure. Energy Decomposition Analysis using Absolutely Localized Molecular Orbitals (ALMO‐EDA) as a function of hydrostatic pressure shows an expected increase in Pauli repulsion between the two fragments, i. e. the metal site and its surrounding ligands, and an increase in favorable charge transfer from orbital interactions that arises due to an amplification of the influence of the ligand field.

Additionally, we find room for precise engineering of the ligand field environment around the metal site with a mixed ligand system, which serves to modulate the spin gap of the metal ion. We demonstrated the possibility of decreasing the spin transition pressure in asymmetric octahedral metal‐ligand complexes, which has wide ramification in gas separation, storage and transport. Such asymmetric octahedral configurations routinely exist in metal‐organic frameworks with open metal sites, which in some cases are pentacoordinated, leaving room for the adsorption of a gas molecule.

## Conflict of interest

The authors declare no conflict of interest.

## Supporting information

As a service to our authors and readers, this journal provides supporting information supplied by the authors. Such materials are peer reviewed and may be re‐organized for online delivery, but are not copy‐edited or typeset. Technical support issues arising from supporting information (other than missing files) should be addressed to the authors.

Supplementary
