# Supplementary material for: Quantum Chemical Modeling of Pressure‐Induced Spin Crossover in Octahedral Metal‐Ligand Complexes
Source: Chemphyschem. 2019 Oct 10;20(21):2742–7. doi: 10.1002/cphc.201900853 (PMC6899727; doi:10.1002/cphc.201900853)
Supplement: Supplementary file 1 — Supplementary [file CPHC-20-2742-s001.pdf]

CHEMPHYSCHEM

Supporting Information

© Copyright Wiley-VCH Verlag GmbH & Co. KGaA, 69451 Weinheim, 2019

Quantum Chemical Modeling of Pressure-Induced Spin Crossover in Octahedral Metal-Ligand Complexes

Tim Stauch[+],* Romit Chakraborty[+], and Martin Head-Gordon© 2019 The Authors. Published by Wiley-VCH Verlag GmbH & Co. KGaA. This is an open access article under the terms of the Creative Commons Attribution License, which permits use, distribution and reproduction in any medium, provided the original work is properly cited.

Supporting Information

Quantum Chemical Modeling of Pressure-Induced Spin Crossover in Octahedral Metal-Ligand Complexes

Prof. Dr. Tim Stauch,*[1,2] Dr. Romit Chakraborty*[2] and Prof. Dr. Martin Head-Gordon[2,3]

[1]*University of Bremen, Institute for Physical and Theoretical Chemistry, Leobener Str. NW2, D-28359 Bremen, Germany*

[2]*Kenneth S. Pitzer Center for Theoretical Chemistry, Department of Chemistry, University of California, Berkeley, California 94720, United States of America*

[3]*Chemical Sciences Division, Lawrence Berkeley National Laboratory, Berkeley, California 94720, United States of America*

* = authors contributed equally

Contents

1 Computational Details S3

2 Deviation from Spherical Symmetry S4

3 Energies and Average Metal-Ligand Distances as a Function of Hydrostatic Pressure S5

4 Energy Decomposition Analysis as a Function of Hydrostatic Pressure S10

5 Energies, $<S^2>$ values and geometries S15

1 Computational Details

Spin transition pressures were obtained by optimizing molecular coordinates under hydrostatic pressure for high spin and low spin states until the spin pairing energy for low spin configurations was offset by the potential energy cost for maintaining a high spin configuration for a given ligand field. At each step of the geometry optimization, the geometries converged when the restoring force of the molecule equaled the hydrostatic compressive force-field (HCFF). Absence of any spurious imaginary frequencies in nuclear modes validated that the converged geometries were indeed true minima. Electronic structure calculations were performed with the ωB97M-V functional, a range-separated hybrid with VV10 correction for non-local dispersion that has been tested extensively for main-group elements at the basis set limit.[1,2] We used the Karlsruhe triply split-valence (def2-TZVP) basis for our calculations.[3] The HCFF model was implemented in and computations were performed with a development version of Q-Chem 5.1.

In the Energy Decomposition Analysis using Absolutely Localized Molecular Orbitals (ALMO-EDA),[4] one ligand was defined as fragment 1 and the rest of the complex as fragment 2. In the case of homogeneous octahedral ligand fields, the choice of the ligand that is defined as a fragment is arbitrary, whereas in the mixed-ligand system described in the main paper ($[Fe(II)(NH_3)_5CO]^{2+}$) the carbon monoxide ligand was defined as fragment 1.

The spherical approximation, inherent in the model, is valid for the systems under consideration within a wide range of pressure, since an octahedral metal complex with a homogeneous ligand field can be projected symmetrically onto a sphere. Since external stresses may distort the octahedral symmetry of complexes under study, calculations were run without constraining the molecule to an octahedral symmetry.

When comparing energy differences between high spin and low spin configurations and the average metal-ligand distances found in the octahedrally coordinated complexes as well as the results from the ALMO-EDA, the calculated hydrostatic pressure is somewhat different between the two spin states, since we estimate the macroscopic pressure by the molecular van-der-Waals surface and this value is generally different for different spin states. However, to facilitate the comparison between high spin and low spin complexes, the calculated macroscopic pressure at the high spin and low spin geometries were averaged in these cases. The spin gaps for the bare metal ions were obtained by averaging over the J values of the relevant spin states in the NIST database.[5]

2 Deviation from Spherical Symmetry

An interesting effect can be observed in the case of metal ions surrounded by six nitrogen molecules (cf. e.g. the middle panel of Figure S1): While the average metal-ligand distance in the low spin case decreases smoothly, the curve for the high spin state jumps at the critical pressure. As a result, the spin-transition occurs abruptly and not, as in the case of the hydrogen and carbon monoxide ligands, smoothly. The reason for this behavior is a change of the ligand coordination in the high spin complex. As the pressure increases, it becomes energetically more favorable for the system to minimize its van-der-Waals surface area by flipping some of the nitrogen ligands from an end-on to a side-on cooordination, because any system under spherically applied forces strives to minimize its surface area. Increasing the pressure further ultimately leads to all nitrogen ligands being in a side-on coordination.

It is important to note the difference between the behavior of the nitrogen, hydrogen and carbon monoxide ligands. The effect described above is only observed in nitrogen, whereas hydrogen remains side-on and carbon monoxide end-on (coordinating with the carbon atom) no matter how high the pressure is. This is because hydrogen has no free electron pairs with which to coordinate to the metal. In carbon monoxide there is a significant amount of back-bonding from the metal to the ligand, which is energetically very favorable, and most metals are low spin when coordinated by carbon monoxide anyway.

3 Energies and Average Metal-Ligand Distances as a Function of Hydrostatic Pressure

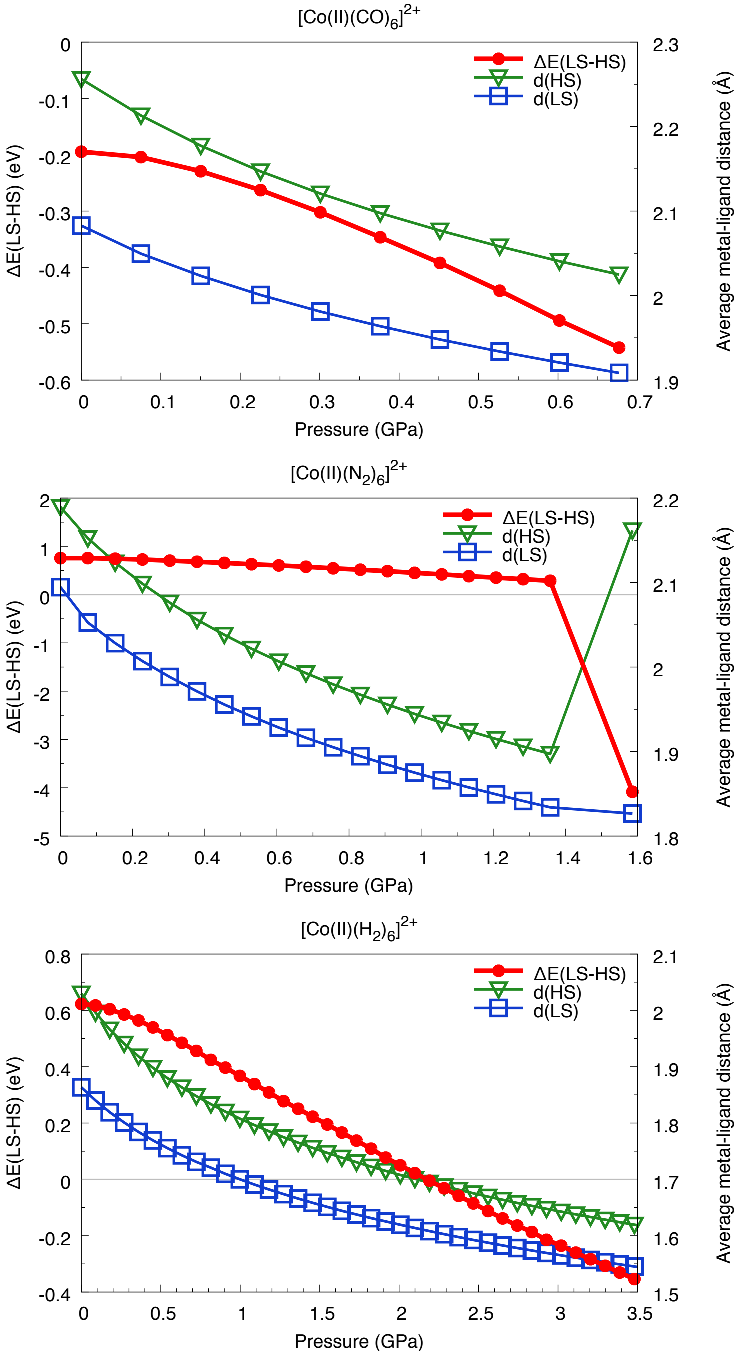

Figure S1: Energy difference between low spin and high spin states in $[Co(II)(CO)_6]^{2+}$ (top), $[Co(II)(N_2)_6]^{2+}$ (middle) and $[Co(II)(H_2)_6]^{2+}$ (bottom). The average metal-ligand distance in the high spin (green) and low spin (blue) states is given on the second y-axis.

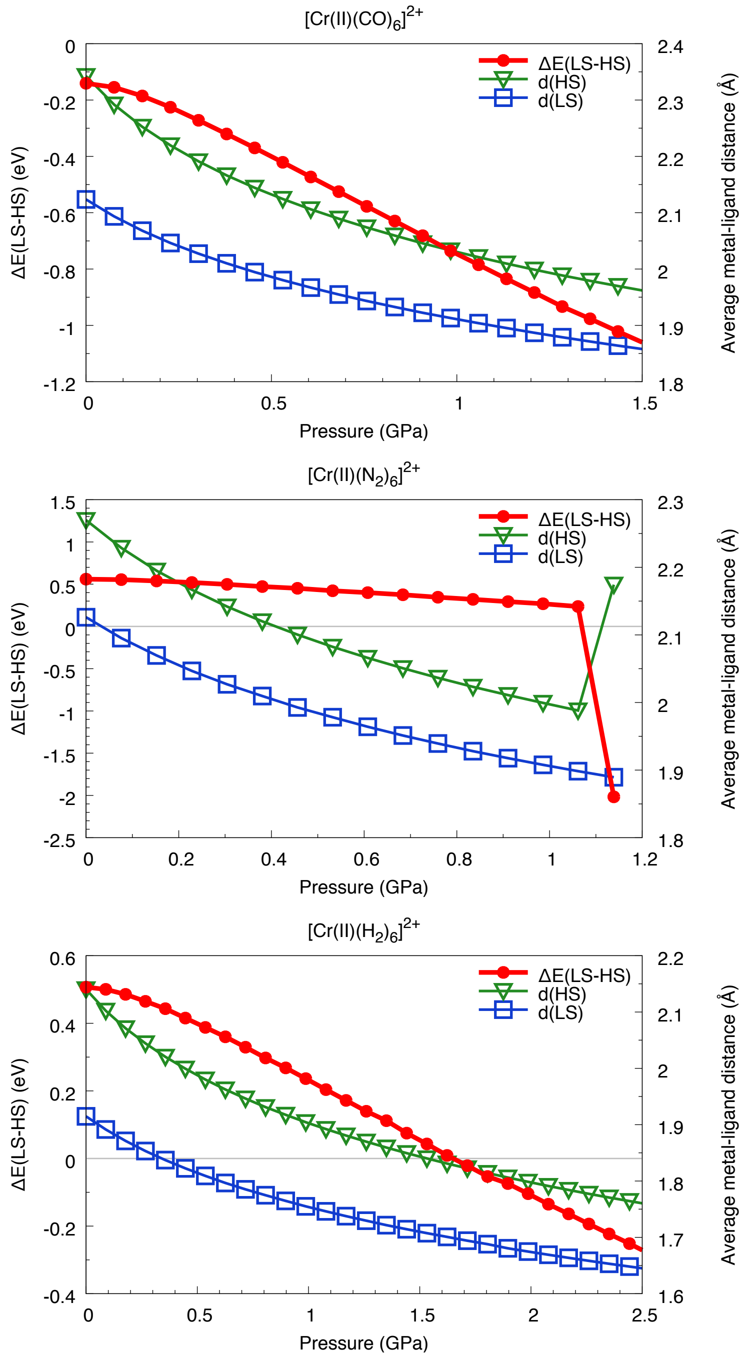

Figure S2: Energy difference between low spin and high spin states in $[Cr(II)(CO)_6]^{2+}$ (top), $[Cr(II)(N_2)_6]^{2+}$ (middle) and $[Cr(II)(H_2)_6]^{2+}$ (bottom). The average metal-ligand distance in the high spin (green) and low spin (blue) states is given on the second y-axis.

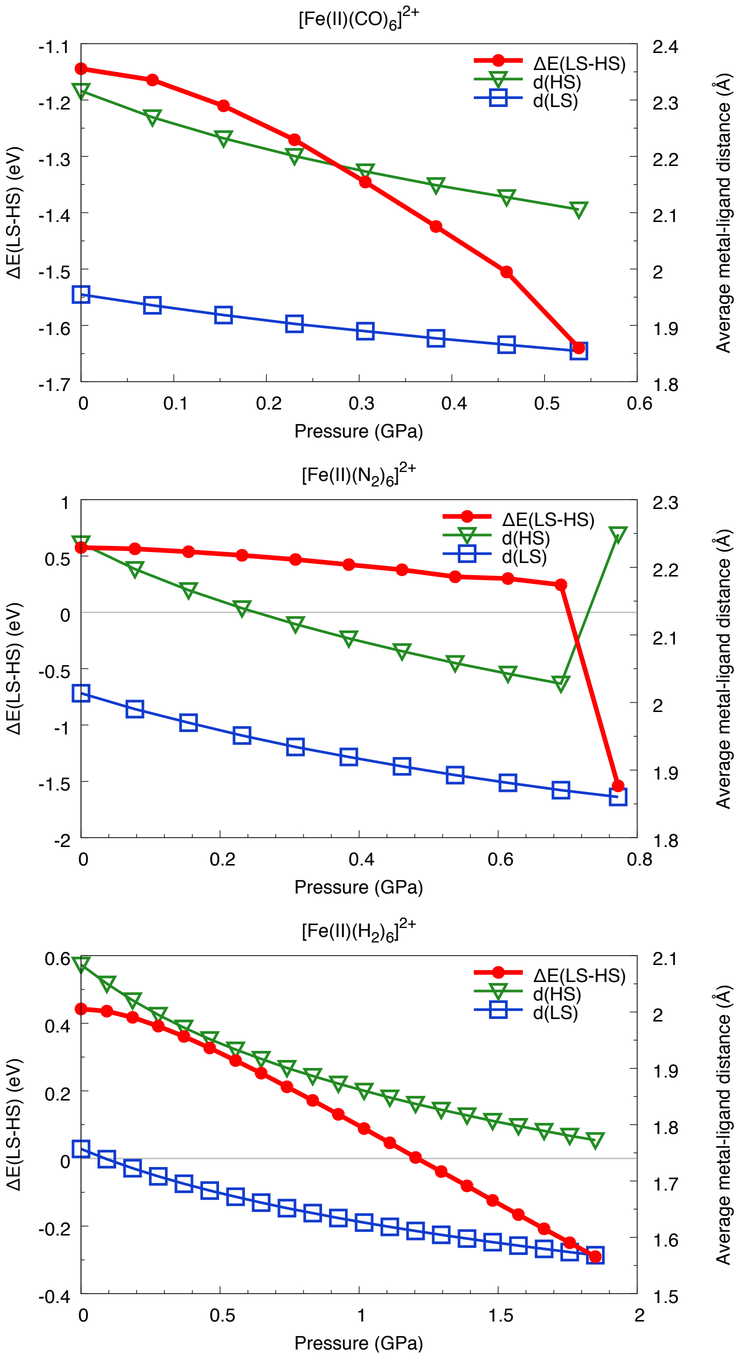

Figure S3: Energy difference between low spin and high spin states in $[Fe(II)(CO)_6]^{2+}$ (top), $[Fe(II)(N_2)_6]^{2+}$ (middle) and $[Fe(II)(H_2)_6]^{2+}$ (bottom). The average metal-ligand distance in the high spin (green) and low spin (blue) states is given on the second y-axis.

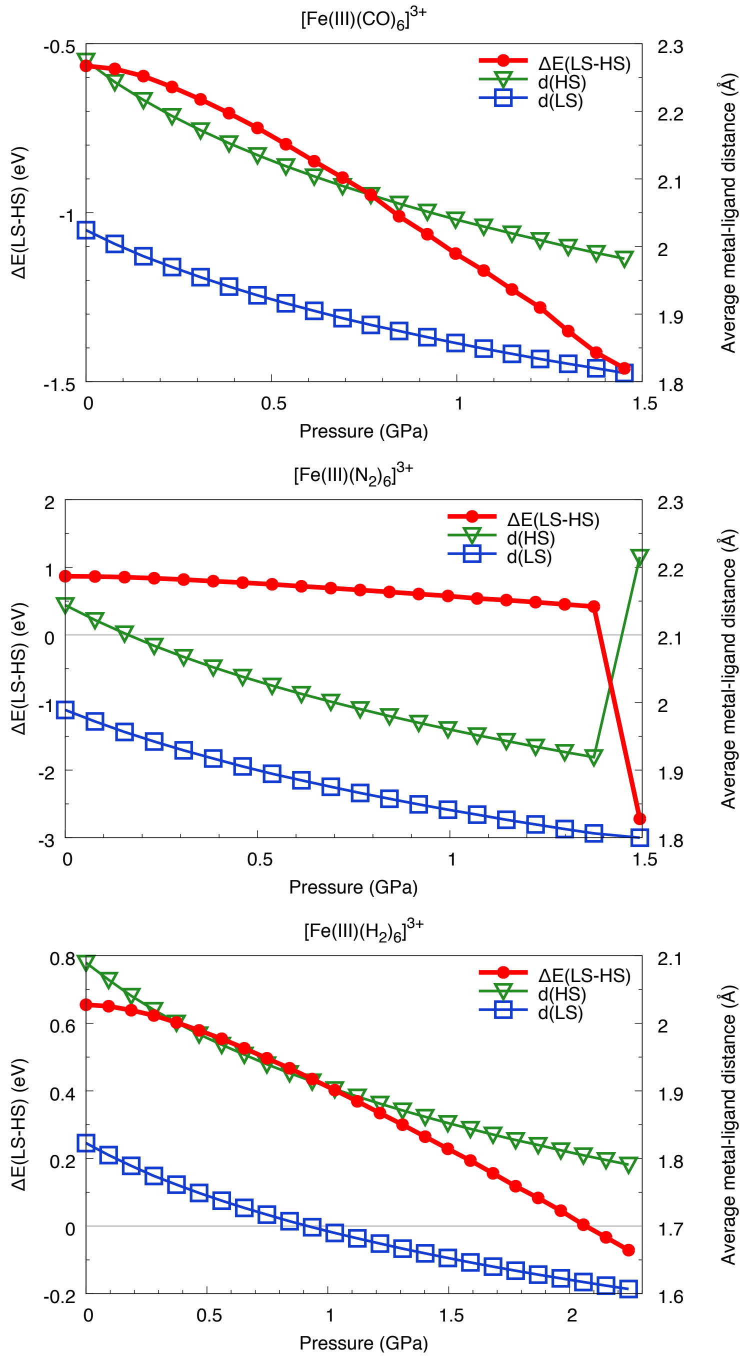

Figure S4: Energy difference between low spin and high spin states in $[Fe(III)(CO)_6]^{3+}$ (top), $[Fe(III)(N_2)_6]^{3+}$ (middle) and $[Fe(III)(H_2)_6]^{3+}$ (bottom). The average metal-ligand distance in the high spin (green) and low spin (blue) states is given on the second y-axis.

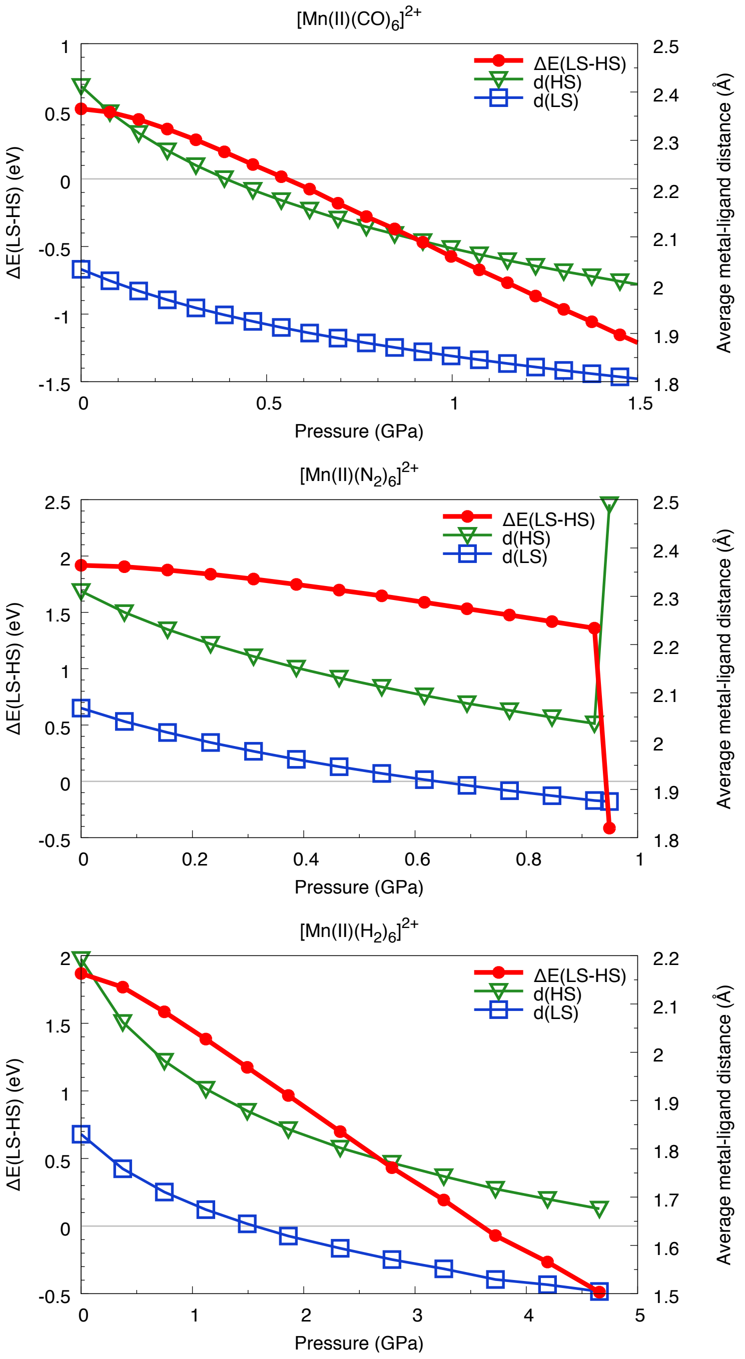

Figure S5: Energy difference between low spin and high spin states in $[Mn(II)(CO)_6]^{2+}$ (top), $[Mn(II)(N_2)_6]^{2+}$ (middle) and $[Mn(II)(H_2)_6]^{2+}$ (bottom). The average metal-ligand distance in the high spin (green) and low spin (blue) states is given on the second y-axis.

4 Energy Decomposition Analysis as a Function of Hydrostatic Pressure

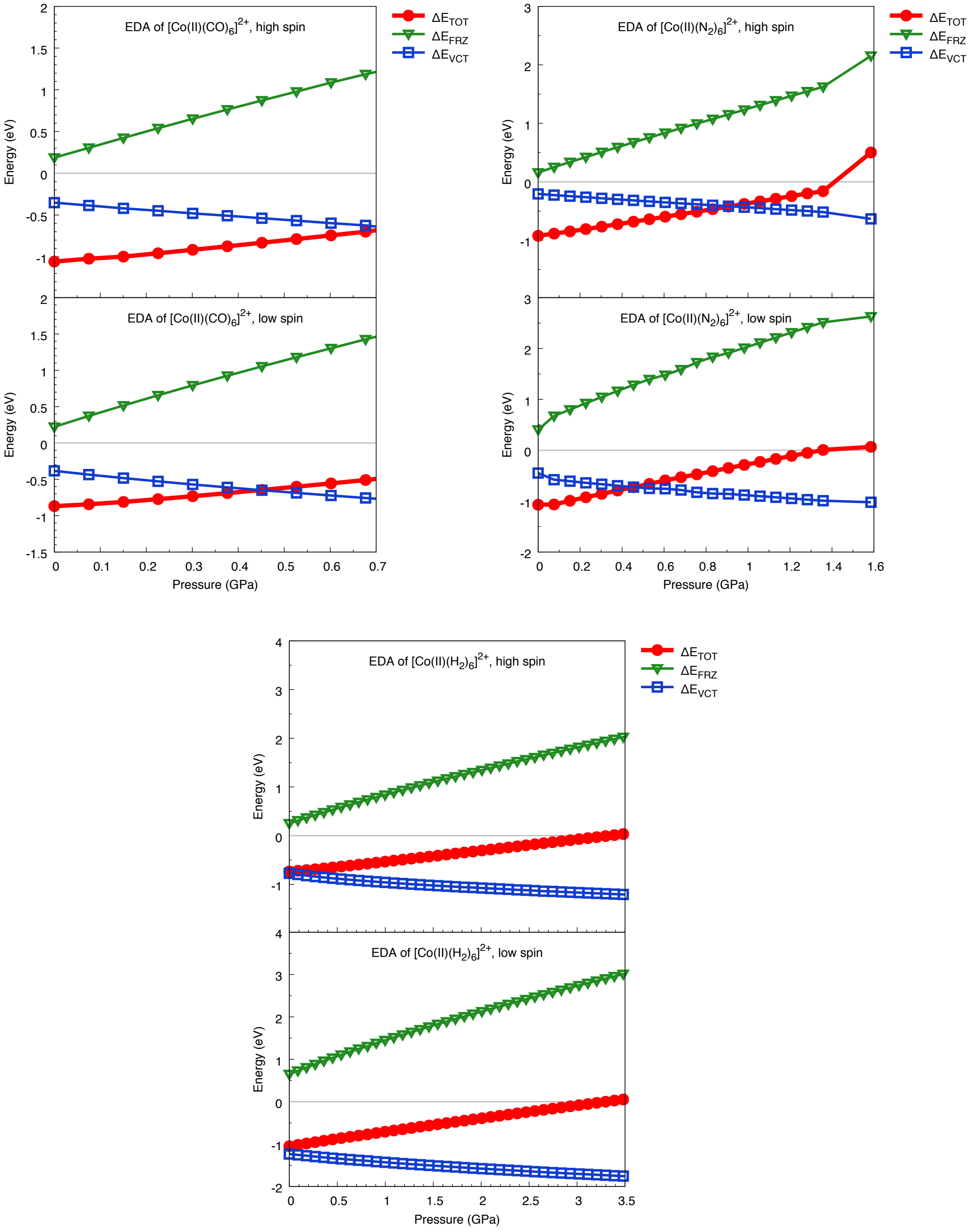

Figure S6: Energy decomposition analysis of contributions to total energy in high spin and low spin in $[Co(II)(CO)_6]^{2+}$, $[Co(II)(N_2)_6]^{2+}$ and $[Co(II)(H_2)_6]^{2+}$. ΔE_{TOT} (red) refers to the total SCF energy, ΔE_{FRZ} (green) to changes in electrostatic repulsion and ΔE_{CT} (blue) to charge transfer as a function of hydrostatic pressure.

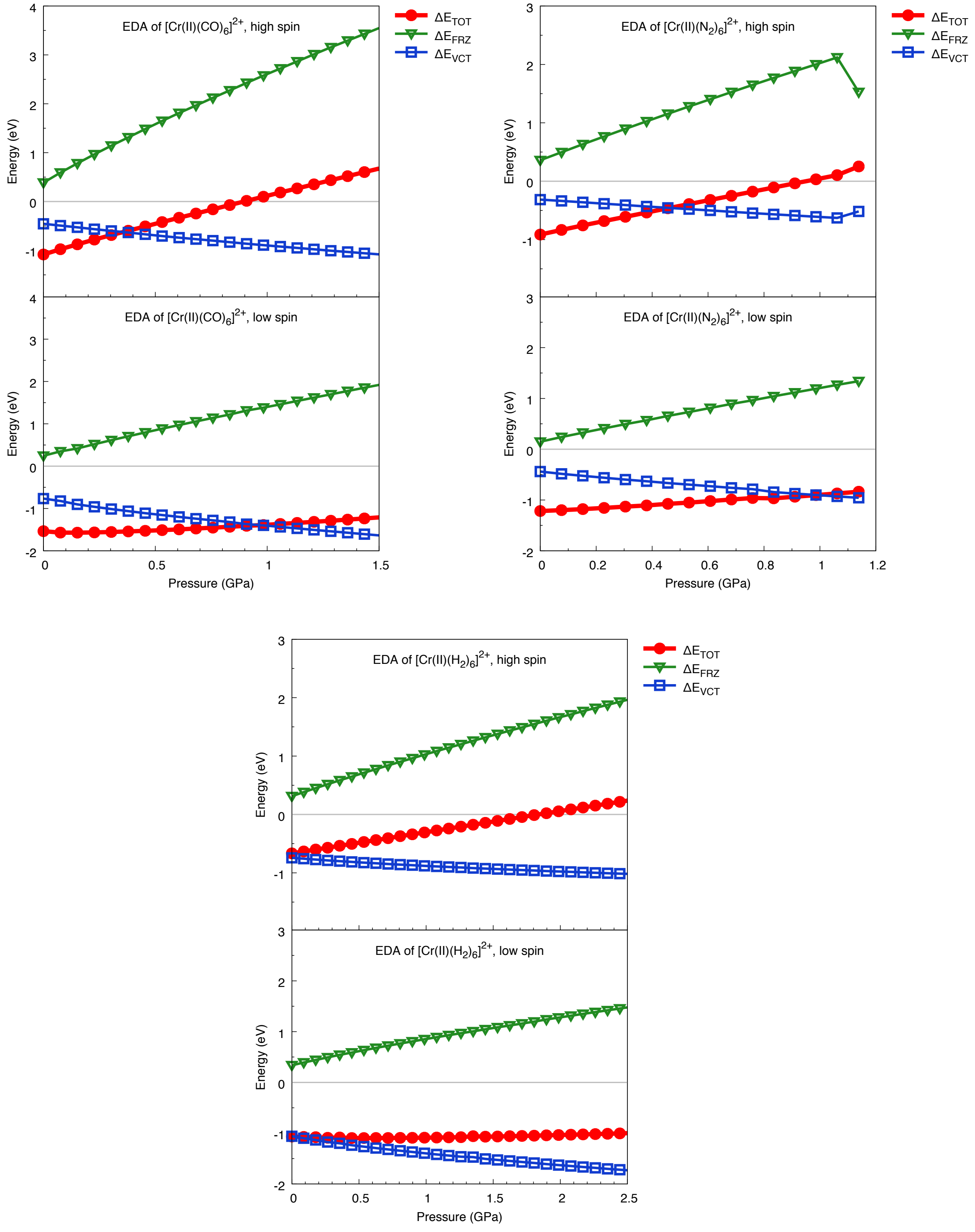

Figure S7: Energy decomposition analysis of contributions to total energy in high spin and low spin in $[Cr(II)(CO)_6]^{2+}$, $[Cr(II)(N_2)_6]^{2+}$ and $[Cr(II)(H_2)_6]^{2+}$. ΔE_{TOT} (red) refers to the total SCF energy, ΔE_{FRZ} (green) to changes in electrostatic repulsion and ΔE_{CT} (blue) to charge transfer as a function of hydrostatic pressure.

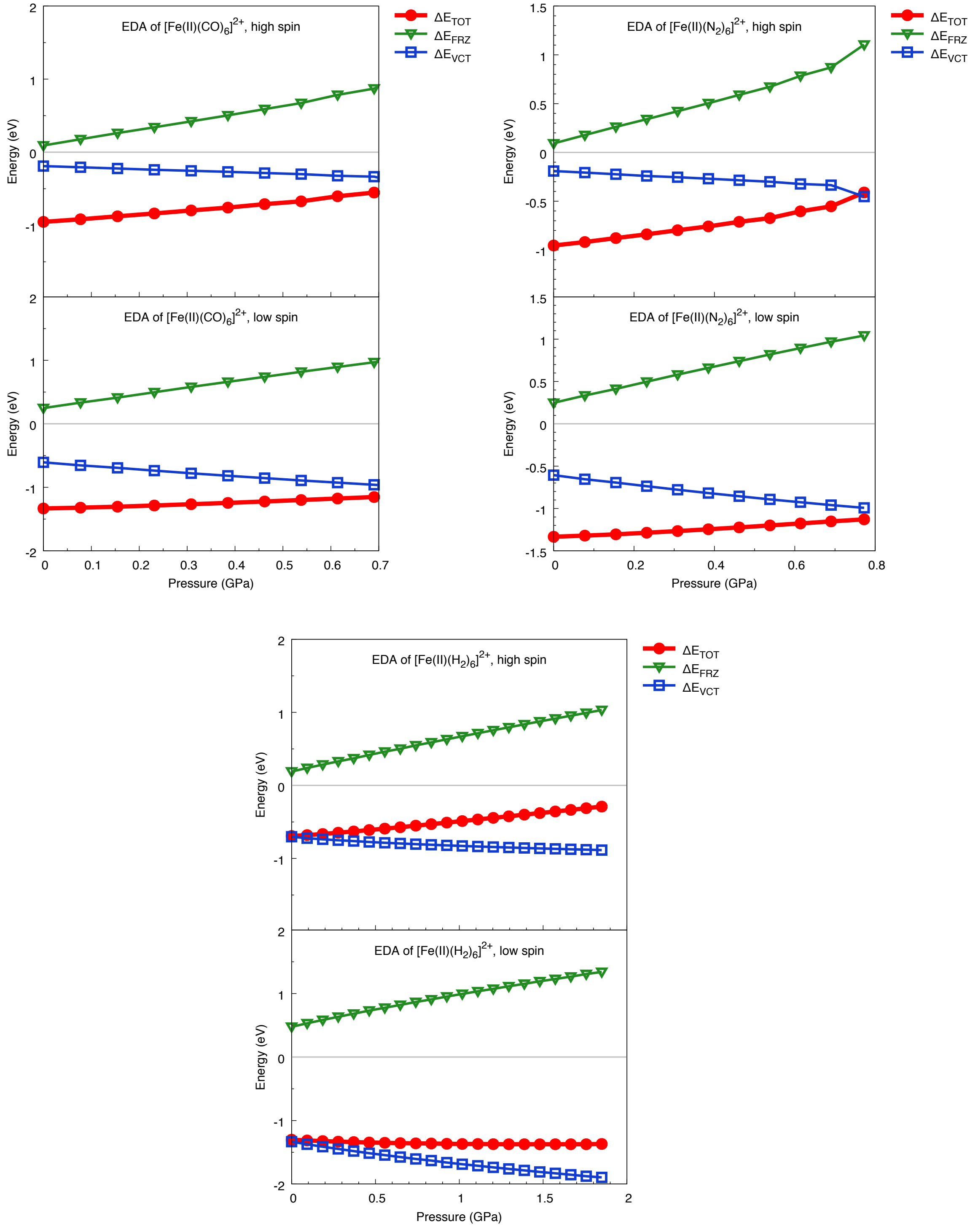

Figure S8: Energy decomposition analysis of contributions to total energy in high spin and low spin in $[Fe(II)(CO)_6]^{2+}$, $[Fe(II)(N_2)_6]^{2+}$ and $[Fe(II)(H_2)_6]^{2+}$. ΔE_{TOT} (red) refers to the total SCF energy, ΔE_{FRZ} (green) to changes in electrostatic repulsion and ΔE_{CT} (blue) to charge transfer as a function of hydrostatic pressure.

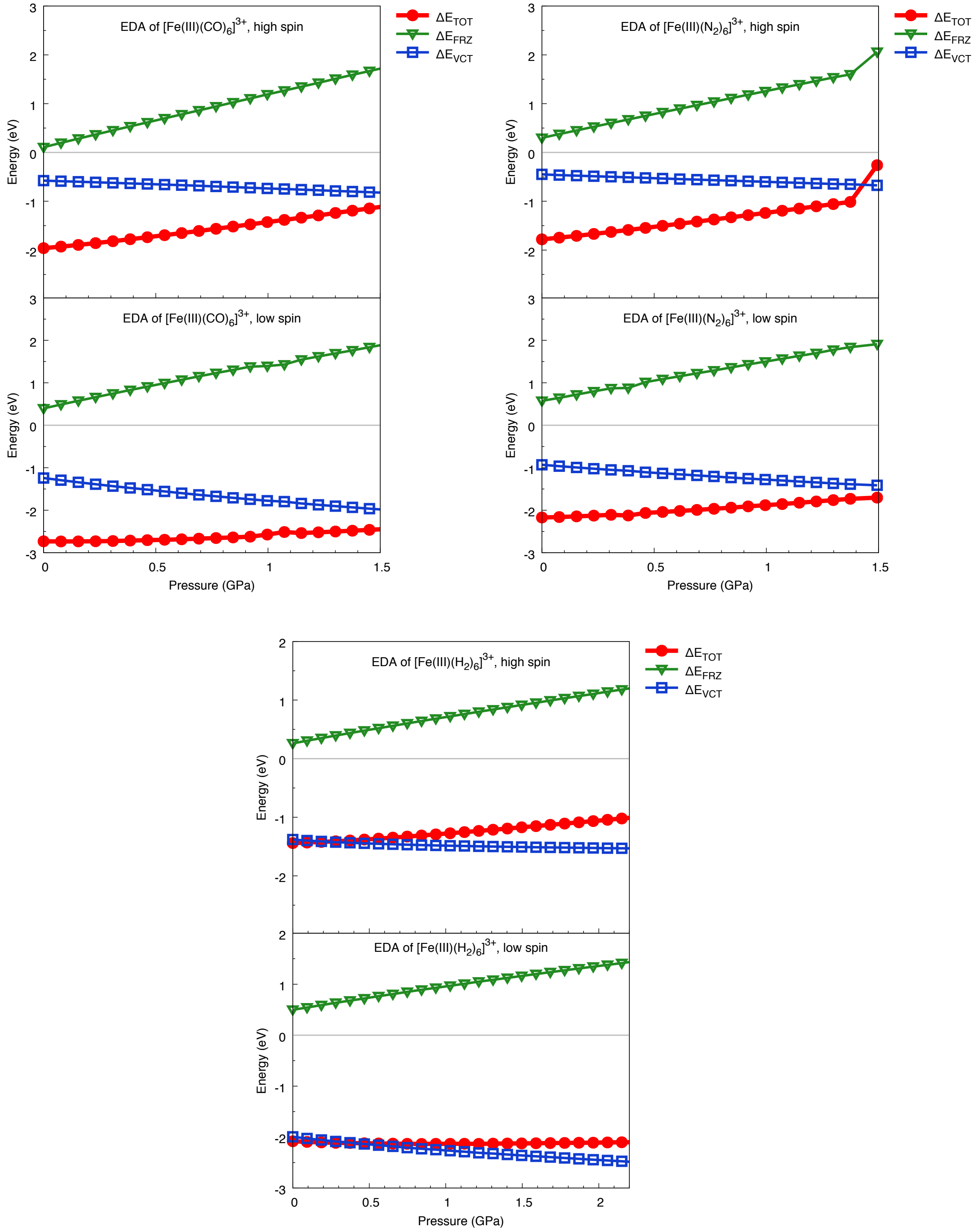

Figure S9: Energy decomposition analysis of contributions to total energy in high spin and low spin in $[Fe(III)(CO)_6]^{3+}$, $[Fe(III)(N_2)_6]^{3+}$ and $[Fe(III)(H_2)_6]^{3+}$. ΔE_{TOT} (red) refers to the total SCF energy, ΔE_{FRZ} (green) to changes in electrostatic repulsion and ΔE_{CT} (blue) to charge transfer as a function of hydrostatic pressure.

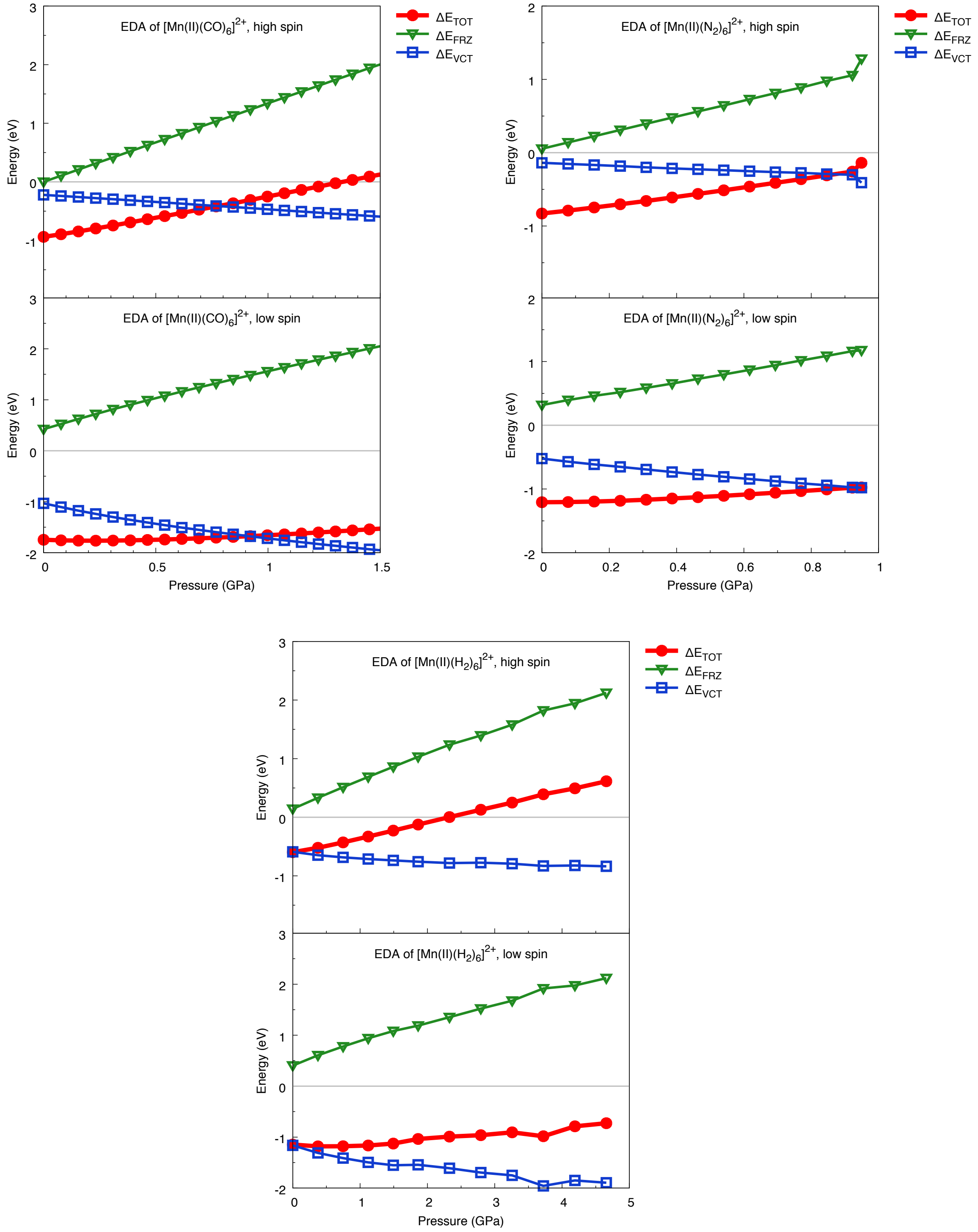

Figure S10: Energy decomposition analysis of contributions to total energy in high spin and low spin in $[Mn(II)(CO)_6]^{2+}$, $[Mn(II)(N_2)_6]^{2+}$ and $[Mn(II)(H_2)_6]^{2+}$. ΔE_{TOT} (red) refers to the total SCF energy, ΔE_{FRZ} (green) to changes in electrostatic repulsion and ΔE_{CT} (blue) to charge transfer as a function of hydrostatic pressure.

5 Energies, <S^2> values and geometries

In the following, the total energies, <S^2> values and geometries (in Å) of the octahedral metal-ligand complexes presented in the paper are given at P=0 and the spin transition pressure. If the metal is already in a low-spin state at P=0, only the values at P=0 are given.

[Co(II)(CO)$_6$]$^{2+}$

P=0

High-spin:

Energy = -2062.14142787345 a.u.

<S^2> = 3.76

Co -0.0004212170 -0.0000950170 -0.0000000767

C -1.6076438517 -0.9672505076 -1.2535253032

C 1.6517332107 -0.8779797312 -1.2607783443

C -0.0548518041 1.8952864120 -1.2233521727

C 0.0553969701 -1.8952161475 1.2235821383

O -0.0849201300 2.8372317729 -1.8123565069

O -2.4089777126 -1.4490371676 -1.8541894133

O 2.4731738703 -1.3130826670 -1.8699522854

O 0.0850387549 -2.8370664446 1.8127519266

C 1.6080906556 0.9667553860 1.2538756173

O 2.4093370819 1.4481586411 1.8550434056

C -1.6514562013 0.8784836359 1.2602917607

O -2.4731819916 1.3140572618 1.8686328602

Low-spin:

Energy = -2062.14859748913 a.u.

<S^2> = 0.76

Co -0.0000538447 0.0001223286 -0.0001034233

C 0.0009160475 1.5441064002 1.2141170095

C 2.3197347723 -0.0012983378 -0.0006736395

C 0.0004507757 -1.2151670399 1.5424795324

C -0.0007967192 1.2157780035 -1.5431011617

O 0.0009712626 -1.9059336719 2.4121203971

O 0.0029539092 2.4141041831 1.9044369786

O 3.4321163941 -0.0022983181 0.0001488305

O -0.0023809613 1.9066555691 -2.4126745392

C -0.0004405474 -1.5427139693 -1.2158246003

O -0.0013660277 -2.4118115470 -1.9072845180

C -2.3198077376 -0.0000134060 0.0021043148

O -3.4321552945 -0.0016478124 0.0042758132

$[Co(II)(N_2)_6]^{2+}$

P=0

High-spin:

Energy = -2039.36320164056 a.u.

$<S^2> = 3.76$

Co 0.0001966575 -0.0000424603 0.0002200528

N 0.2314695097 1.7980431509 -1.2247117941

N 1.6360693959 0.7303002779 1.2571329558

N -1.4863404740 1.0774627128 1.1971720334

N 1.4864210577 -1.0767839193 -1.1980753362

N -2.2232398715 1.6124258243 1.7944053613

N 0.3389524895 2.6851372424 -1.8471384396

N 2.4458244626 1.0911205312 1.8895904045

N 2.2226719856 -1.6115913254 -1.7962531145

N -0.2297212778 -1.7990985106 1.2230391067

N -0.3353986678 -2.6875553824 1.8437887247

N -1.6377590612 -0.7296001771 -1.2548705103

N -2.4497080849 -1.0896966493 -1.8849281669

Low-spin:

Energy = -2039.33526865156 a.u.

$<S^2>$ = 0.75

Co 0.0000968619 0.0006633537 0.0000157319

N 0.0047770997 -1.9940320072 0.0655077255

N 0.0572081511 -0.0653524941 -1.9918613182

N -2.2575036030 -0.0056662483 -0.0573486682

N 2.2574936670 0.0054343327 0.0580532679

N -3.3463884567 -0.0077769438 -0.0773907954

N 0.0078343765 -3.0821810889 0.0943655134

N 0.0843843055 -0.0947338315 -3.0796340537

N 3.3463771116 0.0073170931 0.0794672880

N -0.0029499868 1.9938575726 -0.0663405844

N -0.0038130461 3.0819809513 -0.0951636782

N -0.0591427260 0.0652320828 1.9915630927

N -0.0885917880 0.0950398127 3.0792573461

Spin transition pressure

High-spin:

Energy = -2039.10490189340 a.u.

$<S^2>$ = 3.76

Co -0.0030770617 0.0139678224 -0.0114385935

N 0.4042738404 2.0575011352 -0.5196978690

N 1.0850468895 0.8655800992 1.6423438092

N -1.4059511998 0.4800364266 1.6045620987

N 1.6872417442 -0.4508031509 -1.2619968697

N -1.5883867671 1.2059138479 0.8101882155

N 0.2089332908 1.5791915530 -1.4828699393

N 1.8659023961 0.3259881233 1.1036089913

N 1.1926812927 -1.4155698904 -1.1252790949

N -0.7213620663 -1.8879551920 0.7621790356

N 0.2237964723 -1.7113617264 1.2781031753

N -1.0823609888 -0.5779963300 -1.7770585326

N -1.8671515828 -0.4845909846 -1.0226961122

Low-spin:

Energy = -2039.25505287822 a.u.

$<S^2> = 0.75$

Co -0.0000406122 0.0001404776 -0.0000544599

N -0.0081233179 0.1153196283 1.7849724404

N -0.0547410041 1.7864540850 -0.1154947132

N 1.9012267791 0.0593360194 0.0043560022

N -1.9014643774 -0.0585666330 -0.0045992973

N 2.9777550515 0.0879387291 0.0049236660

N -0.0156318749 0.1810253098 2.8598127742

N -0.0911843433 2.8608155157 -0.1796628851

N -2.9777981319 -0.0924968486 -0.0034865821

N 0.0065725967 -0.1149346352 -1.7851004391

N 0.0068961526 -0.1791586997 -2.8600528075

N 0.0560425397 -1.7860944848 0.1153614873

N 0.1004924333 -2.8601940161 0.1791715540

$[Co(II)(H_2)_6]^{2+}$

<u>P=0</u>

High-spin:

Energy = -1388.92617200172 a.u.

$<S^2> = 3.76$

Co -0.0000955138 0.0000620223 -0.0000882261

H 0.3979998457 -1.5856819613 1.2019938545

H -0.3256514355 -1.7394012179 0.9907549678

H -0.3952950966 1.5827338670 -1.2076239602

H 0.3238483283 1.7428877989 -0.9861152345

H 1.6087063880 -0.4409692993 -1.1675145109

H 1.3480937392 -1.1414187538 -0.9849306489

H -1.3512219011 1.1426162314 0.9797115724

H -1.6068616463 0.4423344598 1.1697723625

H 1.1543993378 1.1536858779 1.2086776544

H 1.6489226895 0.5962586766 1.0179802872

H -1.1560396609 -1.1552402679 -1.2055173330

H -1.6492676424 -0.5954686490 -1.0183680308

Low-spin:

Energy = -1388.90328082805 a.u.

$\langle S^2 \rangle = 0.75$

Co 0.0004850378 0.0001176792 -0.0001825867

H -0.3798472643 1.6960406460 -0.3245356108

H 0.3855691579 1.7211271996 -0.1279254919

H 0.3927253462 -0.2836837311 -1.7004829411

H -0.3895259623 -0.1681443888 -1.7160626846

H 2.0150004094 -0.2498417905 0.2980539315

H 2.0168425182 0.2419717971 -0.2916213207

H -0.3936165937 0.1707773577 1.7146914640

H 0.3892305309 0.2820042493 1.7015250573

H -2.0199371986 0.3127958288 0.2253817090

H -2.0189592259 -0.3088889349 -0.2250388173

H 0.3788022247 -1.7223066835 0.1329721058

H -0.3893799642 -1.6950288894 0.3179724386

Spin transition pressure

High-spin:

Spin transition found Energy = -1388.84999005429 a.u.

$<S^2>$ = 3.76

Co -0.0000232471 -0.0001130853 -0.0000064398

H -0.0307426001 1.4295134043 -0.9084406793

H -0.7619128727 1.1940729697 -0.9296844939

H 0.0305786066 -1.4296264328 0.9085619505

H 0.7618142978 -1.1943539771 0.9295931828

H 1.2158718935 0.7430590042 0.9164166470

H 0.6571006133 1.2702768066 0.9075604944

H -0.6572503553 -1.2703807757 -0.9076384194

H -1.2159654788 -0.7430996220 -0.9164965797

H 1.2496250407 -0.6885327426 -0.9138167850

H 1.4341951376 0.0573205952 -0.9004637817

H -1.2496214963 0.6884219702 0.9138226766

H -1.4341827474 -0.0574315118 0.9005902182

Low-spin:

Energy = -1388.85016847215 a.u.

$<S^2>$ = 0.75

Co 0.0000771162 -0.0000252157 0.0000480131

H -0.3674176090 -0.2429227917 -1.5049408462

H 0.3679797765 0.0777794593 -1.5223161546

H 0.3761492686 -1.5063809128 0.2198223975

H -0.3780755093 -1.5207723006 -0.0545762755

H 1.6434313266 0.2868549657 0.2596134139

H 1.6429034748 -0.2881495792 -0.2613312613

H -0.3772894379 1.5208828503 0.0553743821

H 0.3767182442 1.5061903814 -0.2196177465

H -1.6430214610 0.2507737571 -0.2963339138

H -1.6430492461 -0.2498038092 0.2965019933

H 0.3701265086 -0.0755682185 1.5218194163

H -0.3673455079 0.2405560550 1.5053012084

[Cr(II)(CO)$_6$]$^{2+}$

<u>P=0</u>

High-spin:

Energy = -1723.86205049058 a.u.

$<S^2> = 6.01$

Cr 0.0000242360 -0.0000131016 0.0013635710

C 2.5606698745 0.0014256023 0.0009857271

C 0.0016533176 -2.2168686170 0.2683457441

C -0.0026170683 0.2673373093 2.2174111041

C 0.0027727385 -0.2659361780 -2.2175289542

O -0.0047674218 0.4074959901 3.3192530604

O 3.6739264582 0.0010735126 -0.0000082231

O 0.0026947764 -3.3187126378 0.4087643763

O 0.0050756182 -0.4059002479 -3.3194225981

C -2.5606940373 -0.0028525645 -0.0043571000

O -3.6739443626 -0.0048113936 -0.0077264218

C -0.0018535075 2.2179561055 -0.2659707192

O -0.0030062646 3.3200978382 -0.4041152581

Low-spin:

Energy = -1723.86722762656 a.u.

$<S^2> = 2.02$

Cr 0.0000371622 -0.0005743435 -0.0007234737

C 1.2847556643 0.5829669646 1.5867532334

C 1.2879405521 -1.6613357138 -0.3082813195

C -1.2602673748 -1.1029977124 1.3041652036

C 1.2602134122 1.1033056864 -1.3041026041

O -1.9315024789 -1.6765766575 1.9799093504

O 1.9719867451 0.8861030602 2.4065911594

O 1.9732670626 -2.5220934367 -0.4685753794

O 1.9312473347 1.6770868618 -1.9798528536

C -1.2844377265 -0.5826777689 -1.5872341813

O -1.9710550562 -0.8855769788 -2.4076648826

C -1.2881330276 1.6608654428 0.3088447623

O -1.9740800071 2.5211387807 0.4697545119

[Cr(II)(N_2)$_6$]$^{2+}$

P=0

High-spin:

Energy = -1701.08947130067 a.u.

$\langle S^2 \rangle = 6.01$

Cr -0.0001439318 0.0003926054 0.0004119824

N 0.0080818276 -1.4795037470 1.5968279447

N 0.0088748634 -1.5978385541 -1.4787998759

N -2.4554821819 -0.0117690222 -0.0040995273

N 2.4552415490 0.0132167526 0.0063987647

N -3.5448231465 -0.0168511279 -0.0056028911

N 0.0154263255 -2.2242298022 2.3914887738

N 0.0126808272 -2.3937968189 -2.2221759870

N 3.5445869493 0.0190364539 0.0095282150

N -0.0008918279 1.4781266406 -1.5985519175

N 0.0006082246 2.2211202853 -2.3948622350

N -0.0159043186 1.5980286733 1.4783555627

N -0.0279295660 2.3939377743 2.2216750860

[Cr(II)(N_2)$_6$]$^{2+}$

Low-spin:

Energy = -1701.06890445233 a.u.

$<S^2>$ = 2.01

Cr -0.0000371127 0.0000561397 0.0000809508

N -1.3269712058 -0.7593239892 -1.4776068473

N 1.3209134863 0.7290897154 -1.4980722509

N -1.1167426473 1.8091504113 -0.0174551700

N 1.1165854148 -1.8090934952 0.0174920277

N -1.6791635369 2.7419890343 -0.0276380916

N -2.0126333449 -1.1377684378 -2.2347810722

N 2.0042311701 1.0909624674 -2.2653599538

N 1.6791499356 -2.7418625619 0.0276338804

N 1.3271051219 0.7592714930 1.4775451416

N 2.0125570523 1.1378846929 2.2348047884

N -1.3210117989 -0.7289459207 1.4980867277

N -2.0042575353 -1.0908440531 2.2654300426

Spin transition pressure

High-spin:

Energy = -1700.93221959360 a.u.

$<S^2>$ = 6.01

Cr 0.1906177549 0.1027518841 -0.1599144476

N 1.0644316316 0.5322079914 -2.1048770172

N -1.2663412869 -1.4253944147 -1.1901922510

N 1.1688236020 -1.5678038019 -0.1379811576

N -0.8993160538 1.7023106212 -0.0869573089

N 1.3249210775 -2.5706529852 0.2357935353

N 1.5966372225 1.1759319799 -1.3950983720

N -1.5070641147 -0.4251118397 -1.5561759213

N -1.7779243324 2.2662545294 0.1951460715

N -0.7730949967 -1.0477283718 1.6108147830

N -1.6301544251 -0.4294655438 1.3409088216

N 0.8259755100 1.1030005330 1.8428217790

N 1.6824957794 0.5837162057 1.4057176347

Low-spin:

Energy = -1701.00630943142 a.u.

$<S^2>$ = 2.01

Cr -0.0000018262 0.0000144013 0.0000003807

N 1.1719638532 -0.6805728267 1.3166606839

N -1.1740294656 0.6704885708 1.3199920762

N 0.9960106944 1.6052718913 0.0069779079

N -0.9959696631 -1.6052743184 -0.0069928659

N 1.5558385137 2.5296554242 0.0113812893

N 1.8559528184 -1.0711365530 2.0566827518

N -1.8582421862 1.0532286980 2.0638451910

N -1.5556447185 -2.5297546736 -0.0115400457

N -1.1720426649 0.6805199992 -1.3166256629

N -1.8563090266 1.0708186409 -2.0565342247

N 1.1740721391 -0.6703840011 -1.3199941288

N 1.8584251825 -1.0527944694 -2.0638873661

$[Cr(II)(H_2)_6]^{2+}$

P=0

High-spin:

Energy = -1050.64760716917 a.u.

$<S^2>$ = 6.01

Cr 0.0002328816 0.0001173282 -0.0001667682

H -0.3692204331 2.0044366399 -0.2575960544

H 0.3724207928 2.0198537489 -0.0516584150

H 0.3811039996 -0.2184612374 -2.0066739924

H -0.3780214111 -0.0898504030 -2.0170052399

H 2.2801647472 -0.2610383422 0.2816252529

H 2.2815529945 0.2539303424 -0.2767085692

H -0.3808886926 0.0918506950 2.0161300252

H 0.3788122296 0.2170434066 2.0071521829

H -2.2811894665 0.2940431335 0.2427899645

H -2.2809154610 -0.2908053469 -0.2418564748

H 0.3673101354 -2.0205518303 0.0558700729

H -0.3769514463 -2.0033619177 0.2520128228

Low-spin:

Energy = -1050.62897759857 a.u.

$<S^2> = 2.01$

Cr 0.0000219088 0.0000610907 -0.0001903427

H -0.2113609481 -0.1906962464 -1.8541534199

H -0.2356672718 0.5915526792 -1.7650420665

H 1.9365756588 0.4646103459 -0.0703561716

H 1.9448369348 -0.2510526489 -0.3616094328

H -0.0329996323 1.7748743913 0.6040028037

H -0.1348764121 1.8627114057 -0.1730304254

H -1.8997234595 -0.4326539257 0.4225909854

H -1.9811233808 0.2193599241 0.0164526459

H 0.0038444038 -1.7769227375 -0.6008855485

H 0.1429452910 -1.8623367374 0.1705623719

H 0.2032639431 0.1890942093 1.8549869408

H 0.2639608797 -0.5905703132 1.7607743984

Spin transition pressure

High-spin:

Energy = -1050.59249078240 a.u.

<S^2> = 6.01

Cr 0.0000730050 -0.0000013960 0.0000121599

H 0.3708775481 1.7345055315 0.2248229227

H -0.3661210549 1.7499359595 0.0189551804

H -0.3760266598 -0.2082255638 1.7353241842

H 0.3690667246 -0.0334367040 1.7488894928

H -1.8664409415 -0.2633496910 -0.2712343208

H -1.8668198390 0.2596892858 0.2719074109

H 0.3677151460 0.0356531183 -1.7491588173

H -0.3778658855 0.2082442672 -1.7349832105

H 1.8659957077 0.2800732037 -0.2546917648

H 1.8671956261 -0.2783128025 0.2520763933

H -0.3637529937 -1.7505307277 -0.0199665305

H 0.3742640658 -1.7341358418 -0.2220354337

Low-spin:

Energy = -1050.59255713208 a.u.

<S^2> = 2.01

Cr 0.0001409087 0.0005027688 -0.0005409795

H 0.0139155735 -1.0918890023 -1.2636355453

H -0.0436589551 -0.3704798652 -1.6286373736

H 1.6803525341 0.3979369126 -0.2883959585

H 1.7271722830 -0.2400448667 0.1431414111

H -0.1050861677 1.6264722928 -0.3680164933

H -0.1283954578 1.2593259623 -1.0903447919

H -1.7019385278 0.1607209266 0.3746314806

H -1.7046696391 -0.3242902300 -0.2253575112

H 0.0826515327 -1.6264307936 0.3679176622

H 0.0890270150 -1.2601609910 1.0911612072

H 0.0242092912 1.0944212528 1.2610797817

H 0.0657967307 0.3722467085 1.6267083119

[Fe(II)(CO)$_6$]$^{2+}$

P=0

High-spin:

Energy = -1943.08915243872 a.u.

$<S^2> = 6.01$

Fe 0.0012975869 -0.0001158159 0.0002626914

C 0.0368133008 2.2988121302 0.0416764688

C -0.9257701829 0.0550155018 -2.1108874794

C 2.1545707340 -0.0190230572 -0.9216440253

C -2.1555318720 0.0192313263 0.9221660782

O 3.1795239419 -0.0280986166 -1.3503688812

O 0.0558985916 3.4102856342 0.0605606430

O -1.3794438582 0.0772689332 -3.1256384078

O -3.1800757215 0.0283952432 1.3522259090

C -0.0405516906 -2.2987870167 -0.0418033577

O -0.0609159674 -3.4101207390 -0.0599696485

C 0.9269728770 -0.0550580455 2.1095769928

O 1.3834184815 -0.0774971824 3.1230231307

Low-spin:

Energy = -1943.13122279062 a.u.

$<S^2> = 0.00$

Fe 0.0000509486 0.0000599104 0.0000040497

C -1.4369430524 0.8457782022 -1.0207275093

C -0.6784993691 -1.7619009714 -0.5060246825

C 1.1403056878 0.0158573976 -1.5874421091

C -1.1401230019 -0.0151979408 1.5875246952

O 1.7883563210 0.0253922545 -2.4907220757

O -2.2547731149 1.3261862036 -1.6006256688

O -1.0641853146 -2.7641674562 -0.7934936304

O -1.7880054346 -0.0236933818 2.4909107131

C 1.4356085205 -0.8463746120 1.0221356365

O 2.2505818609 -1.3282455395 1.6048471101

C 0.6797655569 1.7619878228 0.5045530823

O 1.0677743426 2.7642207869 0.7890560552

Spin transition pressure

High-spin:

Energy = -1943.08915243872 a.u.

$<S^2> = 6.01$

Fe 0.0012975869 -0.0001158159 0.0002626914

C 0.0368133008 2.2988121302 0.0416764688

C -0.9257701829 0.0550155018 -2.1108874794

C 2.1545707340 -0.0190230572 -0.9216440253

C -2.1555318720 0.0192313263 0.9221660782

O 3.1795239419 -0.0280986166 -1.3503688812

O 0.0558985916 3.4102856342 0.0605606430

O -1.3794438582 0.0772689332 -3.1256384078

O -3.1800757215 0.0283952432 1.3522259090

C -0.0405516906 -2.2987870167 -0.0418033577

O -0.0609159674 -3.4101207390 -0.0599696485

C 0.9269728770 -0.0550580455 2.1095769928

O 1.3834184815 -0.0774971824 3.1230231307

Low-spin:

Energy = -1943.13122279062 a.u.

$<S^2> = 0.00$

Fe 0.0000509486 0.0000599104 0.0000040497

C -1.4369430524 0.8457782022 -1.0207275093

C -0.6784993691 -1.7619009714 -0.5060246825

C 1.1403056878 0.0158573976 -1.5874421091

C -1.1401230019 -0.0151979408 1.5875246952

O 1.7883563210 0.0253922545 -2.4907220757

O -2.2547731149 1.3261862036 -1.6006256688

O -1.0641853146 -2.7641674562 -0.7934936304

O -1.7880054346 -0.0236933818 2.4909107131

C 1.4356085205 -0.8463746120 1.0221356365

O 2.2505818609 -1.3282455395 1.6048471101

C 0.6797655569 1.7619878228 0.5045530823

O 1.0677743426 2.7642207869 0.7890560552

[Fe(II)(N$_2$)$_6$]$^{2+}$

P=0

High-spin:

Energy = -1920.31651573254 a.u.

$<S^2>$ = 6.01

Fe -0.0004015814 0.0001691470 -0.0005433506

N 0.8046758142 -1.6675550560 -1.2465472097

N 1.6534762020 -0.3376209395 1.4698676788

N 1.2874909367 1.4539809943 -1.1071670143

N -1.2896164852 -1.4541316965 1.1052889163

N 1.9155059547 2.1612754006 -1.6471022197

N 1.1749766811 -2.4833570195 -1.8658292954

N 2.4539208955 -0.4875197182 2.1929462648

N -1.9179891258 -2.1619003952 1.6439323877

N -0.8069409266 1.6609976753 1.2537750671

N -1.1798786789 2.4691386613 1.8816735881

N -1.6492314571 0.3443124149 -1.4745219050

N -2.4448405099 0.4997864305 -2.2019597557

Low-spin:

Energy = -1920.29536621508 a.u.

$<S^2> = 0.00$

Fe 0.0003771410 -0.0004062022 -0.0001528760

N 0.9605782182 1.2529686073 -1.2512397336

N -1.5813611724 -0.0197325970 -1.2446268629

N 0.7814500293 -1.5776921930 -0.9776793491

N -0.7799011933 1.5777126826 0.9784709211

N 1.2039946693 -2.4364649687 -1.4966024791

N 1.4882278879 1.9269305221 -1.9240896903

N -2.4329144180 -0.0295814883 -1.9228744038

N -1.2005098390 2.4369943076 1.4981285990

N -0.9643682363 -1.2485949515 1.2529575504

N -1.4989908047 -1.9151162241 1.9277152370

N 1.5836077161 0.0154427848 1.2425992940

N 2.4384426354 0.0183136710 1.9167706179

Spin transition pressure

High-spin:

Energy = -1920.23312001904 a.u.

$<S^2> = 6.01$

Fe -0.5871992849 -0.4449128600 0.3253798207

N 1.5847215601 -0.2570710897 0.3999330144

N 0.1132687066 0.4412633918 -1.5623057247

N -0.0814579616 -2.1375937984 -0.6892540194

N -0.3309541770 1.6016053081 0.7890100954

N 0.6746017874 -2.4083351085 -1.4207207958

N 2.5816232896 0.1359767795 0.2324783024

N 0.6719617563 1.0149996271 -2.2936808937

N 0.2567711451 2.5018268504 0.6393447282

N -2.3651988484 0.0547674303 -0.4832760522

N -2.6583083420 0.7962648989 -1.2211862553

N -0.3107038411 -0.8008977369 2.2857330081

N 0.4515438188 -0.4968304445 2.9974109340

Low-spin:

Energy = -1920.27035083591 a.u.

$<S^2>$ = 0.00

Fe -0.0003353019 0.0001164868 -0.0003023745

N 1.3855390627 0.4021786617 -1.1875804843

N 1.1958174148 0.1154528242 1.4317696054

N -0.3824847903 1.8217240815 0.1740498306

N 0.3814359053 -1.8214061704 -0.1754910733

N -0.6080103144 2.8759813572 0.2741101733

N 2.1887962073 0.6327490859 -1.8762900221

N 1.8886336938 0.1885802905 2.2606503099

N 0.6064591728 -2.8756465187 -0.2777960642

N -1.3838153610 -0.4065695389 1.1883061353

N -2.1805336680 -0.6493042853 1.8804677647

N -1.1975319512 -0.1109944910 -1.4317366059

N -1.8928865557 -0.1732575996 -2.2594205813

[Fe(II)(H_2)$_6$]$^{2+}$

P=0

High-spin:

Energy = -1269.88018475983 a.u.

$<S^2>$ = 6.01

Fe 0.0006101883 -0.0000905196 -0.0015949391

H 1.3512487404 -0.8793043935 -1.2916624791

H 0.7761547674 -0.7177208887 -1.7749203274

H -0.7525820012 0.7597217379 1.7659757811

H -1.3167846126 0.9464290835 1.2791921036

H -1.6595886724 0.6997960078 -1.0111933107

H -1.2775387192 0.3611280139 -1.5853080093

H 1.2382073101 -0.4344071236 1.5962920711

H 1.6356471376 -0.7492635838 1.0192074282

H 1.2794549114 1.6876641426 -0.1029348755

H 0.6751248901 1.9504326436 -0.4941118294

H -1.2362169864 -1.6339138095 0.5477132278

H -0.7297652949 -1.9890615529 0.0954868588

Low-spin:

Energy = -1269.86391454925 a.u.

$<S^2> = 0.00$

Fe -0.0000025005 -0.0000960925 0.0000006102

H -1.0842584739 1.3711063602 -0.1739847152

H -1.4597395741 0.9145837021 0.3545436410

H 1.0508067798 -1.3405684662 -0.4311066949

H 1.4694457240 -0.9569287018 0.1225640605

H 1.3661078953 1.0641901000 -0.3028245154

H 0.9392667969 1.4666487776 0.2307839188

H -0.9188044860 -1.4490682454 0.3761176239

H -1.3646029266 -1.0948716334 -0.1759888984

H -0.3869858563 -0.2672484279 -1.6925122128

H 0.1167451842 0.3445365448 -1.7186284086

H 0.4237805489 -0.2966836415 1.6789218666

H -0.1510186859 0.2468500651 1.7326464555

Spin transition pressure

High-spin:

Energy = -1269.84489347906 a.u.

<S^2> = 6.01

Fe -0.0002258787 0.0003971854 0.0008827219

H -1.3354778687 -1.1228636264 0.5079797156

H -0.7514986890 -1.6081760832 0.3890201627

H 0.7644183332 1.6149272982 -0.3326177092

H 1.3525395546 1.1315120867 -0.4380883122

H 1.5803619647 -0.8233727026 -0.3534909022

H 1.1253393933 -1.4152808900 -0.1708356092

H -1.1428083737 1.4091185312 0.1086000265

H -1.5949911006 0.8185894811 0.3022709237

H 0.1740894085 0.4393091045 1.8111513345

H 0.7357613370 -0.0640548255 1.7199423269

H -0.2056537700 0.0848955314 -1.8564388081

H -0.7051975930 -0.4565849484 -1.6708688394

Low-spin:

Energy = -1269.84496547654 a.u.

<S^2> = 0.00

Fe -0.0001727185 0.0001734653 0.0001476680

H -0.9242334926 1.2278621380 -0.4783522586

H -1.2714249612 0.9699921536 0.2059433516

H 0.8219395178 -1.3756741328 -0.1406426494

H 1.3231674008 -0.8842806977 0.2623642512

H 1.2695946924 0.7261918059 -0.6783649140

H 0.9148176282 1.3033160116 -0.2354301904

H -0.8192154573 -1.1002284367 0.8403698833

H -1.3170928891 -0.9012152606 0.2340086278

H -0.5765712919 -0.6624328537 -1.3496764323

H -0.0047611217 -0.1471359525 -1.6024760345

H 0.5753145143 0.1341697641 1.4976938949

H 0.0090824651 0.7111069035 1.4440167971

$[Fe(III)(CO)_6]^{3+}$

P=0

High-spin:

Energy = -1942.36580332012 a.u.

$<S^2> = 8.75$

Fe -0.0000496301 -0.0000271150 -0.0000165654

C 0.5715293121 0.9555555798 -1.9830807541

C 1.1740500345 1.6022946856 1.1084683309

C -1.8632081070 1.3026351815 0.0927210920

C 1.8625444257 -1.3033794911 -0.0935287581

O -2.7706719550 1.9362015153 0.1374258624

O 0.8503710960 1.4203265281 -2.9490213064

O 1.7448065912 2.3827442153 1.6486058808

O 2.7696944097 -1.9372196621 -0.1386947226

C -0.5677461946 -0.9568244530 1.9835665337

O -0.8435701976 -1.4223666094 2.9500830840

C -1.1773423196 -1.6004324238 -1.1082803355

O -1.7503390098 -2.3794846724 -1.6482445424

Low-spin:

Energy = -1942.38659137970 a.u.

$<S^2> = 0.77$

Fe 0.0000294810 -0.0000944064 -0.0001159925

C 1.2391648636 -1.1421825986 1.1214861403

C 0.3857295935 1.6490253705 1.1090032593

C 1.6077794918 0.4855868079 -1.1294950402

C -1.6077478058 -0.4860675253 1.1294920059

O 2.4862021917 0.7494856945 -1.7489949957

O 1.9153562075 -1.7667483311 1.7361446620

O 0.5969398251 2.5506718268 1.7152419384

O -2.4863002971 -0.7501605443 1.7487348504

C -1.2397847240 1.1423294538 -1.1200886692

O -1.9167349992 1.7670219909 -1.7337453607

C -0.3851259696 -1.6487029517 -1.1098344694

O -0.5955829626 -2.5498524933 -1.7170451617

[Fe(III)(N_2)$_6$]$^{3+}$

P=0

High-spin:

Energy = -1919.56437354655 a.u.

$\langle S^2 \rangle = 8.75$

Fe 0.0005026146 0.0000375233 0.0005673144

N -1.0028225828 1.7341772126 0.7649177039

N -0.8522603439 0.3641610343 -1.9305858171

N 1.6967649072 1.2042742520 -0.5240091080

N -1.6952274774 -1.2039619931 0.5271124553

N 2.5620939833 1.8177226335 -0.7879326927

N -1.5164033830 2.6175552861 1.1527939360

N -1.2823534152 0.5507965150 -2.9178849840

N -2.5606230424 -1.8168166901 0.7921722872

N 0.9927373490 -1.7400332462 -0.7659801467

N 1.4956447820 -2.6284075849 -1.1564244729

N 0.8617442822 -0.3588785348 1.9294244232

N 1.2990215357 -0.5407639366 2.9144459768

Low-spin:

Energy = -1919.53247004062 a.u.

<S^2> = 0.78

Fe 0.0003222615 -0.0001388777 0.0001307829

N 1.5551299543 0.4852341164 1.1404918444

N 1.2266189802 -1.1127440397 -1.1020581836

N -0.3809093428 -1.6085484550 1.1058251383

N 0.3800813788 1.6091485659 -1.1055714487

N -0.5906689209 -2.4807010548 1.7291168951

N 2.4009614820 0.7482905895 1.7797125672

N 1.8929864873 -1.7209603416 -1.7178529837

N 0.5887313215 2.4823308198 -1.7277949941

N -1.5544586431 -0.4840976466 -1.1424824055

N -2.3990408089 -0.7456378235 -1.7840175172

N -1.2269547405 1.1109966805 1.1031062040

N -1.8933998714 1.7171886836 1.7208663479

Spin transition pressure

High-spin:

Energy = -1919.37316876913 a.u.

<S^2> = 8.76

Fe 0.8459205174 -0.0264059858 0.0344808910

N -0.5746210120 0.4326536649 -1.3902102120

N 1.4680278429 1.7638477623 -0.3469266070

N -0.6658816279 0.9748967326 1.0148015363

N 1.4608781709 -1.2782799878 -1.3036034534

N -1.7103766874 1.1997939584 1.1917797859

N -1.6062565620 0.5418703577 -1.7015827587

N 0.6946977360 2.5098479164 -0.5366642621

N 0.6680703290 -1.7616107384 -1.8774063922

N 0.4979223555 -0.7759044358 2.4980039020

N 1.3309900259 -0.5593442063 1.8272687472

N -0.6763372703 -1.3927959386 0.2877174147

N -1.7335181426 -1.6286203893 0.3018458930

Low-spin:

Energy = -1919.46975969106 a.u.

$<S^2> = 0.76$

Fe -0.0000442755 0.0000536621 -0.0001865306

N -0.3371578821 -1.5026520862 0.9392818065

N -1.2911360231 -0.4800600012 -1.1645078183

N -1.2386291015 0.9104948715 0.9435486363

N 1.2382832145 -0.9106338519 -0.9439611505

N -1.9575081078 1.4386752946 1.5528160245

N -0.5409378111 -2.3693742994 1.5510526493

N -2.0346970612 -0.7555004152 -1.8982019444

N 1.9585238950 -1.4385698406 -1.5518203041

N 0.3370978232 1.5033664285 -0.9384442779

N 0.5414838720 2.3686616334 -1.5520471412

N 1.2916206131 0.4800752901 1.1636265713

N 2.0332698568 0.7553898393 1.8992323388

[Fe(III)(H$_2$)$_6$]$^{3+}$

P=0

High-spin:

Energy = -1269.01968938544 a.u.

$<S^2> = 8.75$

Fe -0.0000307946 0.0000261834 -0.0000590831

H -1.1036411302 -1.2593381713 -1.2484028425

H -1.6407875960 -0.6874204452 -1.0995998318

H 1.1129890343 1.2687850093 1.2314622539

H 1.6337359000 0.6741593407 1.1173473342

H 1.0200094940 1.1362543953 -1.4254129754

H 0.4755411222 0.7728291473 -1.8826796060

H -0.4798383752 -0.7597840521 1.8868438425

H -1.0104572187 -1.1413373190 1.4280724657

H 1.1862975125 -1.7204882172 0.0418369152

H 1.4715171098 -1.3540813257 -0.6079604218

H -1.1941765954 1.7149257524 -0.0485103274

H -1.4705489183 1.3548420558 0.6085983627

Low-spin:

Energy = -1268.99561583942 a.u.

$<S^2> = 0.78$

Fe -0.0000237519 0.0000100546 0.0000282853

H -1.2357323979 -0.9239209766 0.9691198855

H -1.5418940137 -0.1599961638 0.9575784675

H 0.1893067675 -1.5276403411 -0.9778268758

H -0.6257222286 -1.4128379025 -0.9644097375

H -0.8997406667 1.2489722570 -0.9777488113

H -1.4149262140 0.6072061441 -0.9737238348

H 0.6256953378 1.4132099040 0.9644872683

H -0.1894647023 1.5271675798 0.9781624192

H 1.4164508671 -0.6066147590 0.9725599793

H 0.8995375906 -1.2469061599 0.9795631095

H 1.5386899748 0.1574673982 -0.9652640504

H 1.2381909961 0.9237631506 -0.9634287333

Spin transition pressure

High-spin:

Energy = -1268.95200807459 a.u.

<S^2> = 8.75

Fe 0.0004707232 0.0002210850 -0.0007136549

H 1.3096655812 -0.7579125861 -0.9826571765

H 0.8536490147 -0.4346017526 -1.5280198435

H -1.3018705004 0.7674902926 0.9837608399

H -0.8597685753 0.4217939670 1.5268229297

H 0.7011690262 1.6156268646 0.3884488257

H 1.1777704787 1.3549846775 -0.1725972255

H -1.1825348881 -1.3499232868 0.1696118702

H -0.6985549647 -1.6181956353 -0.3814126550

H 0.8084033480 -1.0628788456 1.2110302506

H 1.2343411669 -0.4093247832 1.2494502354

H -0.8067696317 1.0611062940 -1.2146631417

H -1.2375431213 0.4103620361 -1.2463092830

Low-spin:

Energy = -1268.95213325622 a.u.

<S^2> = 0.76

Fe 0.0000003171 -0.0000116846 0.0000040806

H -0.7133903014 -1.1669448898 0.8612992752

H -1.2611225184 -0.5374467872 0.8554917377

H 0.6495253360 -1.2021871912 -0.8633803754

H -0.1708273012 -1.3546747324 -0.8655322449

H -1.0908451415 0.8272513701 -0.8586844640

H -1.3723174348 0.0415298011 -0.8516678974

H 0.1706322917 1.3546814196 0.8655430576

H -0.6496555720 1.2018979225 0.8636878867

H 1.3721858673 -0.0414860365 0.8518977666

H 1.0911398235 -0.8273782069 0.8581795936

H 1.2614282345 0.5377554668 -0.8547861276

H 0.7132065381 1.1668229262 -0.8615655804

[Mn(II)(CO)$_6$]$^{2+}$

P=0

High-spin:

Energy = -1830.40667580171 a.u.

$<S^2>$ = 8.75

Mn -0.0014731218 -0.0002987382 0.0008551583

C 1.7355981430 -0.2133710891 -1.6628889838

C -0.1467334787 2.3646068542 -0.4554785878

C -1.6745230898 -0.4281230012 -1.6820789217

C 1.6754758845 0.4279284338 1.6815472914

O -2.4496541162 -0.6256615425 -2.4542483674

O 2.5357383986 -0.3111538670 -2.4285082219

O -0.2148476738 3.4545384907 -0.6637721808

O 2.4498330107 0.6250692076 2.4545395751

C -1.7336731224 0.2140322662 1.6622881512

O -2.5333402318 0.3120215937 2.4283081480

C 0.1472926541 -2.3643800973 0.4542164726

O 0.2142963750 -3.4544003505 0.6628046110

[Mn(II)(CO)$_6$]$^{2+}$

Low-spin:

Energy = -1830.38762855259 a.u.

$<S^2>$ = 0.76

Mn 0.0000256859 -0.0000764288 -0.0000158209

C -0.7580730362 1.5282309646 1.1048432546

C 0.9559717939 1.4124574949 -1.1061518033

C 1.6914190389 -0.1133929509 1.1218348700

C -1.6915961996 0.1130509559 -1.1215316396

O 2.6210963000 -0.1759252225 1.7284199526

O -1.1752102077 2.3674241762 1.7031615573

O 1.4827512306 2.1871762945 -1.7049918932

O -2.6216292291 0.1753668739 -1.7276508867

C 0.7575980495 -1.5280126476 -1.1059403939

O 1.1740098635 -2.3666885816 -1.7055329633

C -0.9552572690 -1.4125001135 1.1068890972

O -1.4811259018 -2.1870287252 1.7067661470

Spin transition pressure

High-spin:

Energy = -1830.37067193987 a.u.

$\langle S^2 \rangle = 8.75$

Mn -0.0009032858 0.0001947207 -0.0004767745

C 1.5534066947 0.1856759370 1.5063280972

C -0.1558154125 -2.1245513845 0.4219702338

C -1.5129286046 0.4091779197 1.5031164226

C 1.5124116380 -0.4088300240 -1.5036888703

O -2.2860638518 0.6183587066 2.2645547140

O 2.3463696125 0.2788031668 2.2704772400

O -0.2367160258 -3.2060617188 0.6342571000

O 2.2856483399 -0.6176897163 -2.2651371942

C -1.5527917754 -0.1858368254 -1.5062067411

O -2.3450092088 -0.2795698586 -2.2709502581

C 0.1549247065 2.1245253799 -0.4219109674

O 0.2356740741 3.2061196724 -0.6337348687

Low-spin:

Energy = -1830.37082362800 a.u.

$\langle S^2 \rangle = 0.76$

Mn 0.0000188318 -0.0000688270 -0.0000453324

C -0.6949042707 1.4428109236 1.0408856318

C 0.9110639883 1.3170467594 -1.0411585438

C 1.5884115418 -0.1248564832 1.0534467611

C -1.5885666324 0.1245160311 -1.0532145335

O 2.5144348214 -0.1971565189 1.6555760314

O -1.1004756585 2.2831406116 1.6364053559

O 1.4433188796 2.0837495926 -1.6363688575

O -2.5149214518 0.1966513520 -1.6548789777

C 0.6945403367 -1.4426028479 -1.0417626535

O 1.0993662300 -2.2824127711 -1.6385396822

C -0.9104887306 -1.3170779899 1.0417442689

O -1.4418261281 -2.0836245446 1.6379722987

[Mn(II)(N_2)$_6$]$^{2+}$

<u>P=0</u>

High-spin:

Energy = -1807.64183637018 a.u.

$\langle S^2 \rangle$ = 8.75

Mn 0.0016497900 0.0010679361 -0.0013983114

N -0.1304857319 -2.1273222982 -0.8949038615

N -0.8503702267 -0.7924729656 1.9972751154

N 2.1398488152 -0.4390018764 0.7473099724

N -2.1382168445 0.4393570494 -0.7502471171

N 3.1543336652 -0.6447629085 1.0862302986

N -0.1974319034 -3.1302563268 -1.3145378559

N -1.2483861628 -1.1636541865 2.9408049005

N -3.1523805105 0.6447898340 -1.0903618406

N 0.1176303132 2.1283023302 0.8949031746

N 0.1737481275 3.1312359648 1.3161025792

N 0.8614927578 0.7911828926 -1.9945390253

N 1.2672141631 1.1612005175 -2.9351878495

Low-spin:

Energy = -1807.57135577407 a.u.

<S^2> = 0.78

Mn 0.0004806439 0.0000049871 0.0003728752

N 0.7395214172 1.5647376753 -1.1320438304

N -1.6584693944 -0.2059678921 -1.2186459010

N 1.0743657717 -1.3951748864 -1.0858420290

N -1.0743003550 1.3952194285 1.0857250746

N 1.6473958975 -2.1153635864 -1.6682131734

N 1.1273727230 2.3904094310 -1.7270582201

N -2.5253371359 -0.3144826687 -1.8689609860

N -1.6479468477 2.1148303574 1.6681847067

N -0.7404010618 -1.5642707657 1.1320282395

N -1.1280294866 -2.3897650866 1.7274317920

N 1.6587120774 0.2054314356 1.2183117024

N 2.5256893698 0.3143935452 1.8683983220

Spin transition pressure

High-spin:

Energy = -1807.44848717690 a.u.

<S^2> = 8.75

Mn 0.0423115923 -0.0001262684 -0.0017517095

N 1.1361516391 1.7968558254 -1.0290611680

N 1.6490793083 -0.3486471195 1.6359684558

N -1.3008445916 1.2188665009 1.3683506242

N 0.4639373573 -1.7942766255 -1.4586401930

N -1.0447878580 1.9428553413 0.5918580785

N 1.6973275625 0.8729826895 -1.1847138335

N 1.3931774268 0.7133203192 1.6188765066

N 1.1910984554 -1.7829345420 -0.6440048899

N -1.3594822436 -1.6447674239 0.6587959013

N -0.8183355712 -1.4461522794 1.5864718711

N -1.0824429768 0.3517645174 -1.8705295325

N -1.9647614186 0.1196571171 -1.2703039024

Low-spin:

Energy = -1807.52902667904 a.u.

$<S^2> = 0.76$

Mn 0.0000204077 -0.0000279212 0.0000143449

N -1.2212397168 -0.9575641026 1.0542227660

N -0.2731676995 1.5573874990 1.0106302278

N 1.4330459243 -0.5213721208 1.0928244257

N -1.4329668781 0.5213767965 -1.0927782896

N 2.2383037609 -0.8073739504 1.7560624529

N -1.9086118802 -1.4967144846 1.6922068423

N -0.4313743915 2.4325072523 1.6264690018

N -2.2382359092 0.8077426449 -1.7558968024

N 1.2211979873 0.9575331487 -1.0542375479

N 1.9083272757 1.4970080169 -1.6922192676

N 0.2732129890 -1.5575363790 -1.0105544659

N 0.4314022825 -2.4327139551 -1.6263237514

$[Mn(II)(H_2)_6]^{2+}$

<u>P=0</u>

High-spin:

Energy = -1157.20540195956 a.u.

$<S^2>$ = 8.75

Mn -0.0000168388 0.0000568549 -0.0000436165

H -1.2085874739 -0.3132124445 -1.8018882207

H -1.6321040535 0.2159452075 -1.4500587786

H 1.2208279153 0.3260132527 1.7916989791

H 1.6212811360 -0.2328184006 1.4592377648

H 1.4353018323 1.4609024596 -0.7830829980

H 0.9264596065 1.4569691444 -1.3524258878

H -0.9287826338 -1.4468661846 1.3616070660

H -1.4274487896 -1.4682416148 0.7837266029

H 0.9120083468 -1.8509446835 -0.7431150812

H 1.3269376800 -1.3355286040 -1.1244272567

H -0.9194208421 1.8507069361 0.7345853782

H -1.3260517537 1.3356535587 1.1252328452

Low-spin:

Energy = -1157.13672174974 a.u.

$<S^2>$ = 0.76

Mn -0.0000842520 0.0003372357 -0.0003756590

H -1.5530330516 -0.1165241133 0.9620123579

H -1.4038906306 0.6566665421 0.9747792031

H 0.8651208680 -1.2672697802 0.9964044089

H 0.1298880915 -1.5476169093 0.9666449184

H -1.2635934832 -0.8880581362 -0.9791068393

H -0.6616471744 -1.3957351021 -0.9795310958

H -0.1316440461 1.5489760702 -0.9676663312

H -0.8668426467 1.2689127524 -0.9962091357

H 0.6637974867 1.3961860713 0.9788094685

H 1.2649495068 0.8877431806 0.9802954563

H 1.4040606962 -0.6561192842 -0.9771330107

H 1.5539589354 0.1168013680 -0.9621708469

Spin transition pressure

High-spin:

Energy = -1157.04833786947 a.u.

$<S^2>$ = 8.75

Mn -0.0000157419 0.0000829984 0.0000653438

H -0.3736203540 -0.0031922087 1.6775803778

H 0.3732180197 -0.0006639252 1.6776639457

H 0.3924781061 -1.6770758522 -0.0015487805

H -0.3549138402 -1.6854566220 -0.0029949622

H -1.6878722117 0.3560359771 0.0020460650

H -1.6795490560 -0.3933066827 -0.0021570864

H 0.3550323605 1.6854813626 0.0022774704

H -0.3923737652 1.6771586061 0.0005884301

H 1.6796151450 0.3930913151 -0.0049679305

H 1.6877974276 -0.3561922813 0.0052223227

H -0.3731454345 0.0003090859 -1.6776365359

H 0.3736867674 0.0019679696 -1.6775130724

Low-spin:

Energy = -1157.04838143930 a.u.

$<S^2>$ = 0.75

Mn 0.0000373212 -0.0003308810 0.0001596819

H -0.3545019547 0.8890543249 1.2153849705

H 0.4657728614 0.8665776866 1.1850323154

H 0.3611986636 -1.2154481500 0.8870222888

H -0.4592397327 -1.1856137689 0.8699502419

H -1.4529838324 0.2525779286 0.3468330753

H -1.4568033362 -0.2450970276 -0.3361580796

H 0.3615504426 1.2126928331 -0.8894268260

H -0.4588898007 1.1876695032 -0.8661102125

H 1.4550493125 0.3371926827 -0.2537295063

H 1.4548886686 -0.3459054870 0.2438175476

H -0.3680490293 -0.8874485845 -1.2126990009

H 0.4525450473 -0.8694936881 -1.1881912490

$[Fe(II)(NH_3)_5CO]^{2+}$

P=0

High-spin:

Energy = -1659.35232449993 a.u.

$<S^2> = 6.01$

Fe 0.2718486901 -0.0042407811 -0.0009077595

N 0.2507391169 2.2664305718 -0.1584712674

C -2.2037368576 0.0135240220 0.0165711388

N 0.0791966491 0.1139335553 2.2179074746

N 2.5292247941 0.0262465622 -0.0058397512

N 0.0416865506 -0.1612823078 -2.2138415722

N 0.2429625841 -2.2757708362 0.1473578452

H -0.3927894369 0.6540616449 -2.6428272494

H -0.5427601214 -0.9467757460 -2.4958784747

H 2.9262048045 0.6323930819 0.7097863983

H 2.9752186283 -0.8774223966 0.1394474155

H -0.6817836636 -2.7017782779 0.1770372421

H 0.7168688071 -2.7372050493 -0.6271200543

H -0.3427945645 0.9812985641 2.5455105065

H 0.9649164581 0.0437903301 2.7160919629

H -0.5039412178 -0.6236479562 2.6097936668

H 2.9258220718 0.3740157399 -0.8770997332

H 0.7160829743 -2.6266169406 0.9783380684

H 0.9172845364 -0.2917303425 -2.7177959075

H 0.6778712205 2.7339338815 0.6392090173

H -0.6701875493 2.6929431016 -0.2451995631

H 0.7742669785 2.6117273696 -0.9610484132

O -3.3196854763 0.0319977595 0.0177184325

Low-spin:

Energy = -1659.34983218927 a.u.

$<S^2> = 0.00$

Fe -0.1354496538 -0.0009614573 -0.0006393725

N -0.2017914553 1.2375853889 -1.6789668843

C 1.7579902586 0.0190843737 0.0128999694

N -0.1024194675 -1.6623303734 -1.2659176538

N -2.2375530614 -0.0086863566 0.0199993540

N -0.1455389069 1.6764573843 1.2421640385

N -0.1899770875 -1.2684729245 1.6578417187

H 0.3525209223 2.4699841573 0.8413074918

H 0.3165324397 1.5244277782 2.1375139658

H -2.6688005257 -0.8528556616 -0.3532103072

H -2.6330321213 0.0806197954 0.9550780134

H 0.6910079084 -1.7291005112 1.8815059922

H -0.4606091112 -0.8056341748 2.5238784014

H 0.5391301567 -1.5506033400 -2.0497711343

H -0.9926012175 -1.9068623878 -1.6963147747

H 0.2097138725 -2.5180467350 -0.8094103994

H -2.6653390914 0.7487154872 -0.5107071738

H -0.8600615379 -2.0285875048 1.5528596031

H -1.0660005839 2.0417079598 1.4811495804

H -0.7426231864 0.8349227250 -2.4426414738

H 0.7007484098 1.4626978302 -2.0949586660

H -0.6346178244 2.1433613186 -1.5052863993

O 2.8794010168 0.0299635175 0.0248573919

Spin transition pressure

High-spin:

Energy = -1659.34543887251 a.u.

$<S^2> = 6.01$

Fe 0.2007806723 -0.0018574024 -0.0022085752

N 0.2619968969 2.1859331799 0.0764327039

C -2.0619618634 0.0098699726 0.0004245176

N 0.0919948096 -0.1111226144 2.1540251447

N 2.3967744765 0.0505839524 -0.0066663969

N 0.0861785430 0.0411481430 -2.1601380534

N 0.2798731359 -2.1900563628 -0.0783850464

H -0.3023716308 0.9054797143 -2.5235446941

H -0.5010596433 -0.7022724374 -2.5246544118

H 2.7725777674 0.4791649742 0.8331368972

H 2.8489375141 -0.8547410767 -0.0789261871

H -0.6202156971 -2.6569670543 -0.0987441907

H 0.7833239031 -2.5187668691 -0.8959515571

H -0.2436474492 0.7427125368 2.5879540651

H 0.9898910130 -0.3135515253 2.5821183717

H -0.5371846763 -0.8457152927 2.4623985519

H 2.7656666484 0.6051052629 -0.7724850277

H 0.7768416797 -2.5763428990 0.7176383701

H 0.9918361733 -0.0728403964 -2.6042351118

H 0.7927905171 2.5140341060 0.8767234790

H -0.6398536620 2.6467686663 0.1312644521

H 0.7282937293 2.5818687504 -0.7332465598

O -3.1757943816 0.0489137036 0.0038890533

Low-spin:

Energy = -1659.34545591034 a.u.

$<S^2> = 0.00$

Fe -0.1184936894 -0.0018153242 -0.0007862088

N -0.2131608721 1.2016885200 -1.6295495230

C 1.7344538176 0.0195761366 0.0139736875

N -0.1162862968 -1.6121062948 -1.2320542730

N -2.1672259698 -0.0119115528 0.0232499608

N -0.1556860737 1.6275404387 1.2038696829

N -0.2016542827 -1.2327664280 1.6080541875

H 0.3379281651 2.4160618608 0.7969852408

H 0.3006655714 1.4696466257 2.0969762838

H -2.5875360667 -0.8704177232 -0.3198692852

H -2.5451767576 0.1066506838 0.9588392701

H 0.6720451796 -1.6979124350 1.8328254566

H -0.4815643593 -0.7588188934 2.4610526753

H 0.5414755347 -1.5052899519 -1.9981150070

H -1.0109805752 -1.8110225010 -1.6689071504

H 0.1600001180 -2.4707046614 -0.7660968349

H -2.5876503194 0.7286545167 -0.5304650484

H -0.8824204716 -1.9748368898 1.4769855792

H -1.0861199698 1.9698325693 1.4236332392

H -0.7577848010 0.7812148721 -2.3763984652

H 0.6823264130 1.4371712259 -2.0452836201

H -0.6624824686 2.0909712822 -1.4333644700

O 2.8559583269 0.0359802128 0.0276759039

References

[1] N. Mardirossian and M. Head-Gordon, *Physical Chemistry Chemical Physics*, 2014, **16**, 9904–9924.

[2] N. Mardirossian and M. Head-Gordon, *The Journal of chemical physics*, 2016, **144**, 214110.

[3] F. Weigend and R. Ahlrichs, *Physical Chemistry Chemical Physics*, 2005, **7**, 3297–3305.

[4] R. Z. Khaliullin, A. T. Bell and M. Head-Gordon, *The Journal of chemical physics*, 2008, **128**, 184112.

[5] A. Kramida, Yu. Ralchenko, J. Reader and and NIST ASD Team, NIST Atomic Spectra Database (ver. 5.6.1), [Online]. Available: `https://physics.nist.gov/asd` [2019, July 16]. National Institute of Standards and Technology, Gaithersburg, MD., 2018.
